# Comparison of the transcriptomic analysis between two Chinese white pear (*Pyrus bretschneideri* Rehd.) genotypes of different stone cells contents

**DOI:** 10.1371/journal.pone.0187114

**Published:** 2017-10-31

**Authors:** Jinyun Zhang, Xi Cheng, Qing Jin, Xueqiang Su, Manli Li, Chongchong Yan, Xiaoyu Jiao, Daihui Li, Yi Lin, Yongping Cai

**Affiliations:** 1 School of Life Science, Anhui Agricultural University, Hefei, China; 2 Horticultural Institute, Anhui Academy of Agricultural Sciences, Hefei, Anhui, China; Wuhan Botanical Garden, CHINA

## Abstract

Stone cell content is thought to be one of the key determinants for fruit quality in pears. However, the molecular mechanism of stone cell development remains poorly understood. In this study, we found that the stone cell clusters (SCCs) distribution and area in ‘Dangshan Su’ (with abundant stone cells) were higher as compared to ‘Lianglizaosu’ (low stone cell content bud sport of ‘Dangshan Su’) based on the histochemical staining, and the correlations of lignin content with stone cell content and SCC area was significant. The fruits of ‘Dangshan Su’ and ‘Lianglizaosu’ at three different developmental stages (23 and 55 days after flowering and mature) were sampled for comparative transcriptome analysis to explore the metabolic pathways associated with stone cell development. A total of 42444 unigenes were obtained from two varieties, among which 7203 differentially expressed genes (DEGs) were identified by comparison of the six transcriptomes. Specifically, many DEGs associated with lignin biosynthesis were identified, including coumaroylquinate 3-monooxygenase (*C3H*), shikimate O-hydroxycinnamoyltransferase (*HCT*), ferulate 5-hydroxylase (*F5H*), cinnamyl alcohol dehydrogenase (*CAD*) and peroxidase (*POD*), as well as genes related to carbon metabolism, such as sorbitol dehydrogenase-like (*SDH-like*) and ATP-dependent 6-phosphofructokinase (*ATP-PFK*). At the peak of the stone cell content (55 days after flowering), the expression level of these genes in ‘Dangshan Su’ was significantly increased compared with ‘Lianglizaosu’, indicating that these genes were closely related to stone cell development. We validated the transcriptional levels of 33 DEGs using quantitative real-time polymerase chain reaction (qRT-PCR) analysis. The results were consistent with the transcriptome analysis, indicating the reliability of transcriptome data. In addition, subcellular localization analysis of three DEGs in lignin synthesis (*PbC3H*, *PbF5H* and *PbPOD*) revealed that these proteins are mainly distributed in the cell membrane and cytoplasm. These results provide new insights into the molecular mechanism of stone cell formation.

## Introduction

*Pyrus bretschneideri* cv. Dangshan Su is a pear variety originating in China that is known for its fruit, which bears a wealth of nutritional and medicinal value. ‘Dangshan Su’ has relatively high stone cell content in the fruit, which is a crucial factor influencing the quality of pear fruit by affecting not only sucrose and other nutrient contents but also flesh hardness and chewiness [1,2]. The ‘Lianglizaosu’ variety originated from a natural bud sport of ‘Dangshan Su’ pear. Years of observation of the trait stability of the two varieties revealed that although the genetic backgrounds are basically identical, the stone cell content of the fruit of ‘Lianglizaosu’ is lower than that of ‘Dangshan Su’ [3]. Therefore, ‘Lianglizaosu’ is thought to be an ideal material to study the developmental mechanism of pear stone cells.

Stone cells are peculiar cells in pear fruit. During the development of pear fruit, stone cells are mainly formed between 23 and 67 days after flowering (DAF) [4]. At present, although the molecular mechanisms related to stone cell development remain unclear, a large number of physiological studies have revealed that stone cells in pear are a form of sclerenchyma cells [4–7]. These cells differentiate from the parenchyma cells of the flesh, and secondary cell wall (SCW) thickening of parenchyma cells and lignin deposition represent key steps in stone cell formation [5–7]. Electron microscopy revealed that a large amount of lignin was transported from the extracellular layer to each layer of the SCW during the stone cell development until the cells were filled [5–8]. Completely developed pear stone cells contain 20–30% lignin. Therefore, lignin is an indispensable component for the development of stone cells [8–12].

Lignin is a biological polymer derived from the dehydrogenative polymerization of three different monolignols, coniferyl alcohol, sinapyl alcohol and p-coumaryl alcohol, forming guaiacyl units (G-units), syringyl units (S-units) and hydroxyphenyl units (H-units), respectively [11,12]. Lignin in pear fruit is mainly composed of G- and S-units [4,13]. The precursors of these two units are coniferyl alcohol and sinapyl alcohol. The synthetic pathway of these alcohols has been elucidated in model organisms in the field of lignin research, such as *Arabidopsis thaliana*, *Populus trichocarpa* and *Eucalyptus grandis* [12,14,15]. The upstream pathway of lignin metabolism is the general phenylpropanoid pathway [16], which mainly involves three enzymes: phenylalanine ammonia-lyase (PAL), cinnamate 4-hydroxylase (C4H) and 4-hydroxycinnamate-CoA ligase (4CL). These enzymes convert L-phenylalanine (L-Phe) to p-coumaroyl-CoA [10,12]. Then, p-coumaroyl-CoA enters into the ester intermediary pathway, which results in the formation of various hydroxycinnamic acids and coenzyme A-thioesters by HCT, C3H and cafeoyl-CoA O-methyltransferase (CCoAOMT) [12,16]. Subsequently, feruloyl-CoA enters the monolignol-specific biosynthesis pathway [17] and forms coniferyl alcohol through a two-step reaction catalyzed by cinnamoyl-CoA reductase (CCR) and CAD [18,19]. F5H and caffeic acid 3-O-methyltransferase (COMT) are responsible for the conversion of coniferyl alcohol to sinapyl alcohol, and POD and laccase (LAC) are responsible for the polymerization of lignin monomers [12,20,21]. However, most of the genes related to lignin metabolism in pear are members of gene families, and it is not clear which family members are critical to lignin synthesis, transport and deposition in stone cells.

In recent years, the completion of pear genome sequencing and the development of genome sequencing technology have greatly promoted genome-wide transcriptome analyses of the formation mechanisms of various traits in pear [22,23]. Genome-wide transcriptome analysis is useful for rapidly analyzing the expression patterns of members of each gene family and understanding the regulatory network of various metabolic pathways. At present, although transcriptome analysis has been used to analyze pear peel color, flower bud and aroma formation and other aspects [24–27], transcriptome analysis of stone cells formation has not been reported.

In this study, ‘Dangshan Su’ and ‘Lianglizaosu’ were selected to explore the metabolic changes and key genes involved in pear stone cell development. Through comparative transcriptome and expression pattern analyses of the two varieties, many genes related to lignin metabolism and some genes associated with carbon metabolism were identified. These results provided new insights into the molecular mechanism of stone cell formation and laid a foundation for clarifying the mutation mechanism of low stone cell content bud sports.

## Materials and methods

### Plant materials

Thirty-year-old ‘Dangshan Su’ (*Pyrus bretschneideri* cv. Dangshan Su) and ‘Lianglizaosu’ (bud sport of ‘Dangshan Su’ pear) trees in the Center of Pear Germplasm Resources, Dangshan County, Anhui Province, China, were selected as seed parents, and ‘Cuiguan’ (*Pyrus pyrifolia*) was used as a pollen parent. All varieties were maintained under the same water-fertilizer regimen and management plan during the years of cultivation. After manual removal of the stamens, artificial pollination was performed by placing pollen on the stigmas of flowers on the branches with 3^rd^, 4^th^ and 5^th^ order flowers (the remaining flowers were removed) during the pear blooming period. After pollination, the stigmas were immediately covered by bags for 7 days. We referred to ‘Cuiguan’ (pollen parent) × ‘Dangshan Su’ (seed parent) as CD and ‘Cuiguan’ (pollen parent) × ‘Lianglizaosu’ (seed parent) as CL.

Previous reports demonstrated that pear stone cell development occurs from 23 DAF to 67 DAF [4]. Therefore, equally sized pear fruits were sampled starting from 23 DAF. A total of 8 developmental periods were sampled: 23 DAF, 39 DAF, 47 DAF, 55 DAF, 63 DAF, 71 DAF, 87 DAF and the mature period (145 DAF). The second sampling interval of 16 days (d) was selected because early pear fruit growth and development is slow. Thereafter, samples were taken every 8 d. Stone cells are completely developed at 67 DAF [4], so the sampling interval is 16 d after the 71 DAF. Finally, ripe fruit (145 DAF) was harvested. Fruits of each period were collected from four directions of the tree (east, south, west and north). Fresh fruits were used for sectioning and microscopy, and the fruits stored at -80°C were used for molecular experiments.

### Observation of stone cell clusters (SCCs)

The morphology of the SCCs was observed according to the method of Cai et al. (2010) [4]. Transverse and longitudinal sections of pear fruits were manually prepared and stained with 1.0% phloroglucinol and 1.0 M hydrochloric acid as described in the Wiesner lignin staining method [28]. The size and distribution of SCCs were observed after chromogenesis.

### Determination of stone cell content

The stone cell content of the fruit was determined using a previously described method [4]: Pulp (5.0 g) was frozen at -20°C for 24 h and then homogenized at 20,000 rpm for 3 min. The homogenized pulp was incubated in water, and the upper suspension was decanted. This procedure was repeated several times. The collected stone cells were oven-dried and weighed. The stone cell content was calculated as follows: stone cell content (%) = weight of stone cells (g DW) / weight of pulp (g FW) × 100.

### Lignin content determination

The lignin content was measured using the method reported by Syros [29] with a few adjustments. Pulp powder (0.02 g) from different developmental stages was collected and placed into a 10-mL frosted glass test tube. Then, 2 mL of 25% bromoacetyl-glacial acetic acid was added, and the tube was sealed with a glass plug. After the mixture was reacted in a 70°C water bath for approximately 30 min with shaking every 10 min, the reaction was terminated by the addition of 3 mL of 2 M NaOH. The liquid mixture was transferred to a volumetric flask, and the volume was adjusted to 100 mL with glacial acetic acid. The absorbance value (ABS) of the solution was measured at 280 nm with three repetitions.

### Statistical analyses

Statistical Program for Social Sciences (release 19.0, SPSS Inc, IBM, www.ibm.com) and Microsoft Excel 2007 were used for the statistical analyses, including standard error and significance analyses (* and ** indicate *P* < 0.05 and 0.01, respectively).

### Sample selection for transcriptomic analysis

Fruit samples from CD and CL at 23 DAF, 55 DAF and the mature period (145 DAF) were selected for transcriptomic analysis. Samples were flash-frozen in liquid nitrogen and pulverized, and 100 mg of each sample was added directly into an RNAse-free microcentrifuge tube containing 1.0 mL of TRIzol Reagent (Invitrogen, USA) and stored at -80°C.

### RNA extraction

RNA was isolated from the pear pulp using a total RNA isolation kit (Tiangen, China). Then, RNA of fruits (in the same period) from four directions was equally mixed for transcriptome sequencing and qRT-PCR analysis. The total RNA was quantified using a NanoDrop 2000 spectrophotometer (Thermo Scientific, USA) by measuring the absorbance ratio of A_260/280_ and A_260/230_, and the integrity was detected by 1% agarose gel electrophoresis. Reverse transcription was performed using the PrimeScript RT Reagent Kit with gDNA Eraser (Perfect Real Time) (Takara, China).

### Library construction, Illumina sequencing and read assembly

High-throughput RNA sequencing (RNA-seq) library construction and sequencing were performed on the Illumina HiSeq 2500 sequencing platform following the manufacturer’s protocols (Illumina Inc., USA). NEBNext Poly (A) mRNA Magnetic Isolation Module (NEB, USA) was used to enrich mRNA, and then, the cDNA library was constructed using the NEBNext mRNA Library Prep Master Mix Set for Illumina (NEB, USA) and NEBNext Multiplex Oligos for Illumina (NEB, USA). The size of the library insert fragments was determined by 1.8% agarose gel electrophoresis, and the fragments were quantified using a Library Quantification Kit/Illumina GA Universal (Kapa, USA). Three biological replicates were used to minimize sample differences.

To obtain clean and high-quality reads for sequence assembly, the raw reads were filtered by removing adapter sequences and low-quality sequences (reads with ambiguous bases ‘N’ or reads containing greater than 50% bases with Q ≤ 10). The Trinity assemble program was used to assemble the clean reads into contigs, which covered more full-length transcripts over a broad range of expression levels [30]. The resultant contigs were added to transcripts based on paired-end information. The longest transcript from alternative splicing transcripts was selected as the unigene. These unigenes were combined to produce the final assembly and used for annotation.

The RNA-seq data has been deposited in Sequence Read Archive (SRA) under accession numbers SRR5965146, SRR5965147, SRR5965142, SRR5965143, SRR5965144 and SRR5965145. Database homepage: https://www.ncbi.nlm.nih.gov/sra.

### Functional annotation

To annotate unigenes, sequences were searched by BLAST against the National Center for Biotechnology Information (NCBI) database to identify the most descriptive annotation for each sequence [31]. The assembled unigenes were compared with sequences in the NCBI non-redundant (nr) protein and nucleotide (nt) databases, including the Swiss-Prot protein database, the Kyoto Encyclopedia of Genes and Genomes (KEGG) and the Cluster of Orthologous Groups (COG) database. Gene Ontology (GO) annotations, including molecular functions, biological processes and cellular components, were also analyzed [32,33]. All searches were performed with an E-value < 10^−5^. Fragments per kilobase of transcript per million fragments mapped (FPKM) was calculated to represent the expression abundance of the unigenes [34]. FPKM may reflect the molar concentration of a transcript by normalizing it for RNA length and for the total read number.

### Differentially expressed gene (DEG) analysis

Gene expression levels were measured in RNA-Seq (Invitrogen) analyses as numbers of reads and were normalized with FPKM [34]. IDEG6 software was used to identify differentially expressed genes in pairwise comparisons, and the results of all statistical tests were corrected for multiple testing; the Benjamini-Hochberg false discovery rate (FDR < 0.01) was used to adjust the *P*-values. FPKM values were obtained by deep sequencing analysis. The ratio represents the fold change in the FPKM value in different development stages: a ratio ≥ 1.2 indicates that genes were significantly up-regulated, and a ratio ≤ 0.8 indicates that genes were significantly down-regulated. Pear genome data were downloaded from the website (http://gigadb.org/dataset/100083) [22].

### Quantitative real-time PCR analysis

To validate the results from the transcriptome sequencing data, the relative expression levels of 33 selected genes were confirmed by qRT-PCR. Primers are listed in S1 Table. Gene quantification was performed using SYBR Green Master Mix (Takara, Otsu, Japan) according to the manufacturer’s instructions. Reactions were performed in a CFX96 Touch™ Real-Time PCR Detection System (Singapore). Each 20 μL reaction mixture consisted of 6.4 μL of nuclease-free water, 10.0 μL of SYBR Premix Ex Taq II, 0.8 μM of each primer, and 2 μL of diluted cDNA. The PCR amplifications were performed as follows: 95°C for 3 min, followed by 40 cycles of 95°C for 10 s, 52°C for 15 s and 72°C 30 s. Each qRT-PCR analysis was performed in triplicate, and a negative control (without template) was included in each reaction. Relative expression levels were calculated using the 2^-ΔΔCt^ method following the protocol of Livak [35]. In this study, *Tubulin* (accession No. AB239680.1) was used as an internal reference [36].

### Subcellular localization of candidate genes

The coding sequences (CDS) of candidate genes (*PbF5H*, *PbC3H* and *PbPOD*) were amplified using primers containing specific enzyme cleavage sites (S1 Table) designed according to the multiple cloning sites within binary vector pCAMBIA1304 (GenBank: AF234300.1). The constructed eukaryotic expression plasmids pCAMBIA1304-*PbF5H*/*C3H*/*POD* were introduced into *Agrobacterium* EHA105 by electroporation. The infection solution with an OD_600_ value of 0.8 was injected into *Nicotiana benthamiana* leaves, and the infected leaves were cultured under dark conditions for 3 days. Microscope slides of infected tobacco leaves were prepared, and green fluorescent protein (GFP) fluorescence was observed with a confocal laser microscope (OLYMPUS, Japan).

## Results

### Stone cell clusters distribution and area in CD and CL fruits

**Fig 1:**
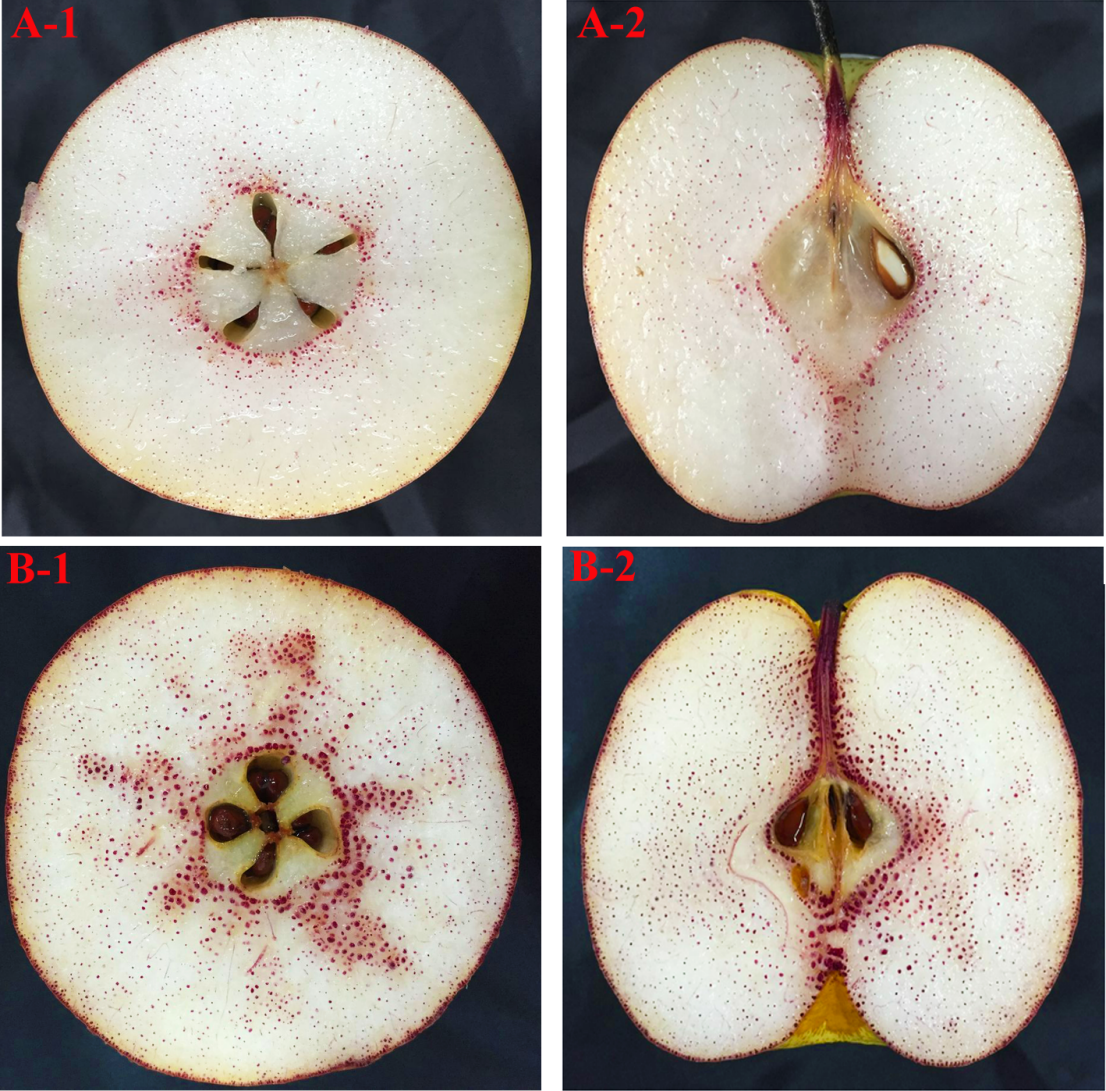
The distribution of stone cell clusters of CD and CL fruit. (A-1): ‘Lianglizaosu’♀×‘Cuiguan’♂ (CL); Cross sections; (A-2): ‘Lianglizaosu’♀×‘Cuiguan’♂ (CL); Longitudinal sections; (B-1): ‘Dangshan Su’♀×‘Cuiguan’♂ (CD); Cross sections; (B-2): ‘Dangshan Su’♀‘Cuiguan’♂ (CD); Longitudinal sections.

After staining them with phloroglucinol, we found that the density and distribution ranges of stone cell clusters (SCCs) in CD fruits were significantly higher than those in CL fruits, regardless of whether cross sections or longitudinal sections were observed (Fig 1).

**Fig 2:**
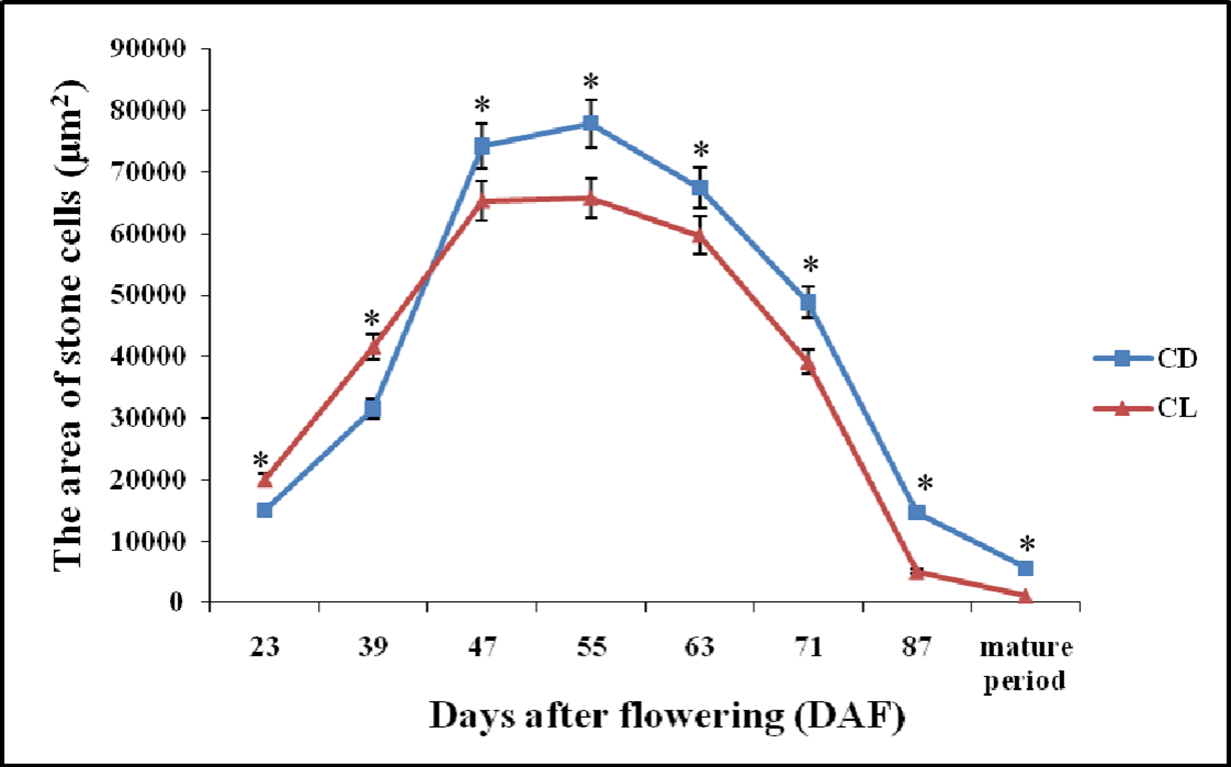
The area of stone cell clusters in CD and CL fruits from different developmental periods. Error bars indicate the standard deviation of five replications. * Significant difference between the stone cell cluster area levels of the two pear varieties in the same period (*P* < 0.05).

**Fig 3:**
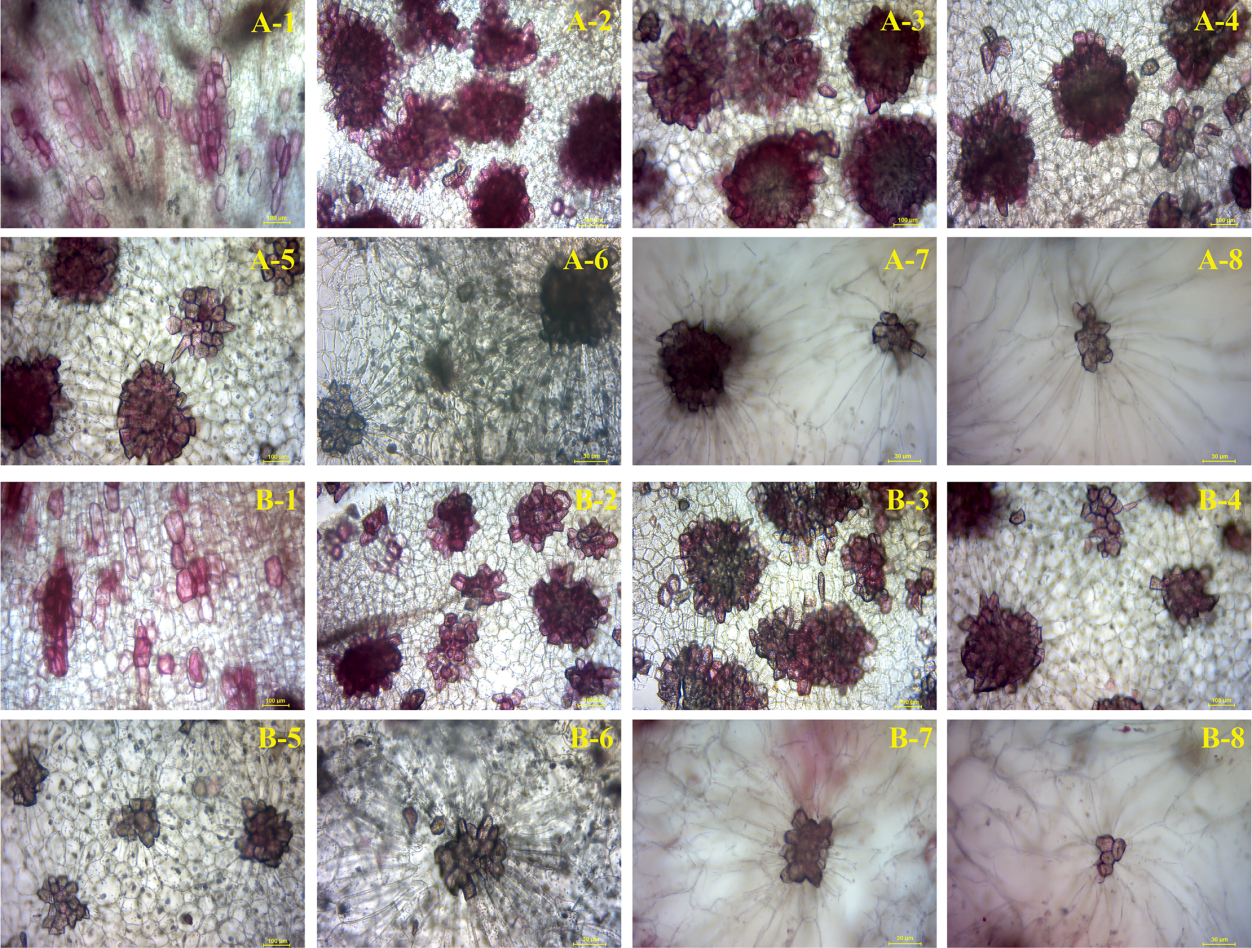
Microscopy of stone cell cluster development in CD and CL fruit. A-1 to A-8 represent the stained sections of the stone cell clusters of CD fruit in eight periods (23 DAF, 39 DAF, 47 DAF, 55 DAF, 63 DAF, 71 DAF, 87 DAF and the mature period); B-1 to B-8 represent the stained sections of the stone cell clusters of CL fruit in eight periods (23 DAF, 39 DAF, 47 DAF, 55 DAF, 63 DAF, 71 DAF, 87 DAF and the mature period).

In both ‘Dangshan Su’ and ‘Lianglizaosu’, the area of SCCs initially increased and subsequently decreased over the course of fruit development (Figs 2 and 3). At 23 DAF, the major SCCs in the fruits of CD and CL are in the primitive stage with loose stone cell aggregation. The SCC area of the two varieties increased rapidly from 23 to 39 DAF. However, after 39 DAF, the cluster area of CD fruits was significant higher compared with that of CL fruits in each period. At 47 DAF, the diameter of the SCCs in the fruits of CD and CL continued to increase until a maximum was achieved at 55 DAF. During the four developmental periods (63, 71, 87 and 145 DAF) after 55 DAF, the SCC areas of the two pear varieties exhibited a decreasing trend, and the areas decreased to the lowest level at maturity. The decrease in area of the SCCs at the later developmental stage may be attributed to the effects of pectinase and cellulase, resulting in degradation of SCCs.

### Divergent content of stone cells and lignin between CD and CL fruits

**Fig 4:**
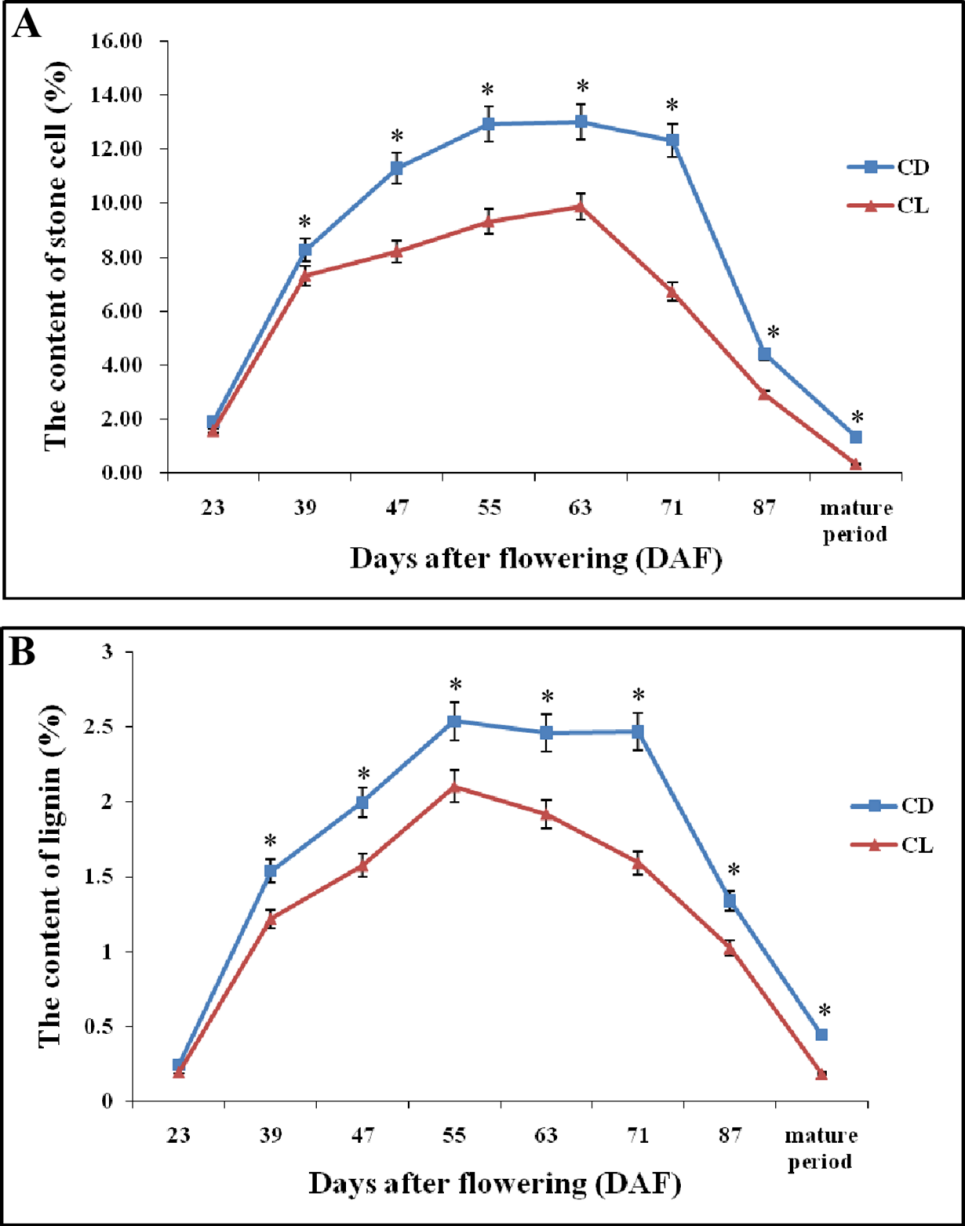
Stone cell and lignin contents during CD and CL fruit development. (A): Stone cell content in different developmental stages. (B): Lignin content in different developmental stages. Error bars indicate the standard deviation of five replications. * Significant difference between the stone cell or lignin levels of the two pear varieties in the same period (*P* < 0.05).

Although the stone cell content of CD fruits over the course of development is higher than that of CL, their trends are very similar (Fig 4A). Stone cell content increased continuously from 23 DAF to 63 DAF. After 63 DAF, the stone cell content declined, reaching its lowest level in the mature stage.

Previous studies have demonstrated that the synthesis and deposition of lignin are closely related to the development of stone cells [4,5,8,13]. In this study, the dynamic changes in lignin content in both CD and CL fruits at different developmental stages were compared (Fig 4B). The lignin contents in CD and CL fruits also increased and decreased at early and later developmental stages, respectively, similar to the stone cell pattern at different developmental stages (Fig 4A). CD fruits achieved their highest lignin content at 55 DAF and 71 DAF, whereas CL fruits contained the most lignin at 55 DAF. Correlation analysis revealed a positive correlation of lignin content with stone cell content and the area of SCCs at different developmental stages (Table 1). The correlation coefficients (r) of lignin content versus stone cell content and SCC area were r = 0.957 (*P* < 0.01) and 0.814 (*P* < 0.05), respectively, in CD fruits and r = 0.951 (*P* < 0.01) and 0.757 (*P* < 0.05), respectively, in CL fruits. This finding indicates that the stone cell content and the area of SCCs of pear were significantly correlated with lignin content.

**Table 1:** Correlation coefficients of lignin content with stone cell content and stone cell cluster area.

	Lignin content of CD fruits (%)	Stone cell cluster area of CD fruits (μm^2^)	Lignin content of CL fruits (%)	Stone cell cluster area of CL fruits (μm^2^)
**Stone cell content of CD fruits (%)**	0.975**	—	—	—
**Lignin content of CD fruits (%)**	1	0.814*	—	—
**Stone cell content of CL fruits (%)**	—	—	0.951**	—
**Lignin content of CL fruits (%)**	—	—	1	0.757*

*, **, Significant at *P* < 0.05 and 0.01.

### Overview of the transcriptome

**Fig 5:**
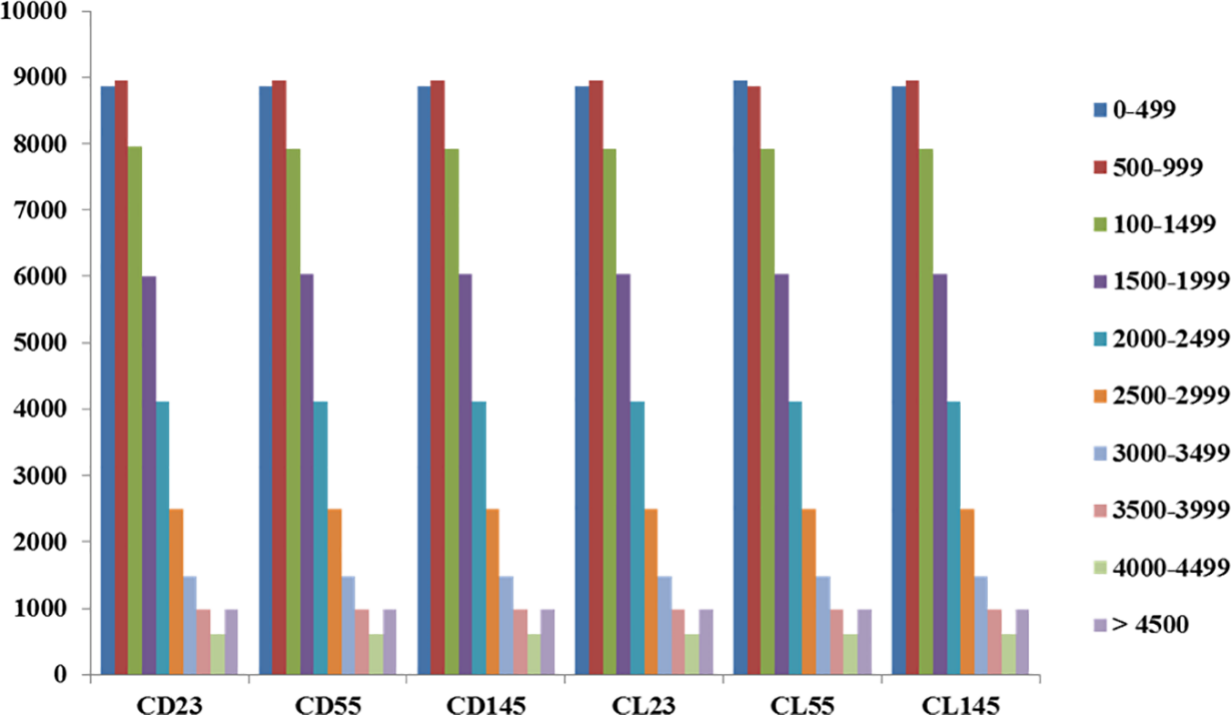
Length distribution of unigenes in the assembled transcriptomes. The colored blocks indicate the lengths of unigenes calculated in 6 libraries. The library names are noted on the X axis, and the number of unigenes are indicated on the Y axis.

As noted in Figs 2 and 4, the SCC area, stone cell content and lignin content of the two varieties reached a high level at 55 DAF, and the difference between the varieties was significant. Furthermore, stone cell development mainly started at 23 DAF, and the content of stone cells was reduced to a very low level in the mature fruit (145 DAF) [4]. The content of stone cells changed significantly during the three periods of the stone cell formation process. Therefore, we selected fruits of the two varieties at 23, 55 and 145 DAF for comparative analysis. A total of six cDNA libraries were sequenced: CD23 (CD at 23 days after flowering), CD55 (CD at 55 days after flowering), CD145 (CD at 145 days after flowering), CL23 (CL at 23 days after flowering), CL55 (CL at 55 days after flowering) and CL145 (CL at 145 days after flowering). After removing the adapters and low-quality sequences, 378,952,686 sequence reads were obtained (Table 2). The GC content of each library was approximately 50%, and Q30% was greater than 96.60% for each library. Thus, the quality and accuracy of the sequencing data were sufficient for further analysis. In addition, most of the reads matched pear genomic locations, and the uniquely mapped reads and multiple mapped reads matched previously described sequences with greater than 56.5% and 8.5% coverage, respectively. The reads mapped to ‘+’ and reads mapped to ‘-’ both matched previously described sequences with greater than 32% coverage. The length distribution of unigenes exhibited similar patterns among the 6 libraries, suggesting minimal bias in the construction of the 6 cDNA libraries (Fig 5).

**Table 2:** Summary statistics for pear genes based on RNA-seq data.

ID	CD23	CD55	CD145	CL23	CL55	CL145
**Raw Reads**	32,062,497	32,926,123	33,896,985	34,778,349	32,776,563	30,262,717
**Clean Reads**	30,563,728	32,129,556	33,037,276	34,105,639	31,868,247	29,776,449
**GC Content**	47.75%	48.28%	48.24%	48.41%	48.36%	48.33%
**%≥Q30**	96.81%	96.65%	96.82%	96.71%	96.74%	96.74%
**Mapped Reads**	71.01%	70.42%	71.69%	70.94%	71.41%	70.93%
**Unique Mapped Reads**	56.79%	61.32%	62.84%	57.92%	60.70%	62.19%
**Multiple Mapped Reads**	14.23%	9.11%	8.85%	13.02%	10.71%	8.74%
**Reads Mapped to '+'**	32.37%	32.63%	33.47%	32.37%	32.95%	33.12%
**Reads Mapped to '-'**	32.38%	32.62%	33.37%	32.41%	32.97%	33.01%

### Unigene annotation and classification

To annotate the unigenes, reference sequences were searched using BLASTX against six databases (NCBI nr database, NCBI nt database, Swiss-Prot, COG, GO and KEGG) (E-value < 10^−5^) (S1 File). A total of 39,533 of 42,444 unigenes yielded a BLAST result (S1 File). S2 File indicates the species with the closest match for each unigene. Most of the annotated sequences exhibited the greatest homology with *Prunus persica* sequences (68.22%), followed by *Fragaria vesca* (10.25%).

### Differing expression of phenylpropanoid metabolism-related genes in CD and CL fruits

**Fig 6:**
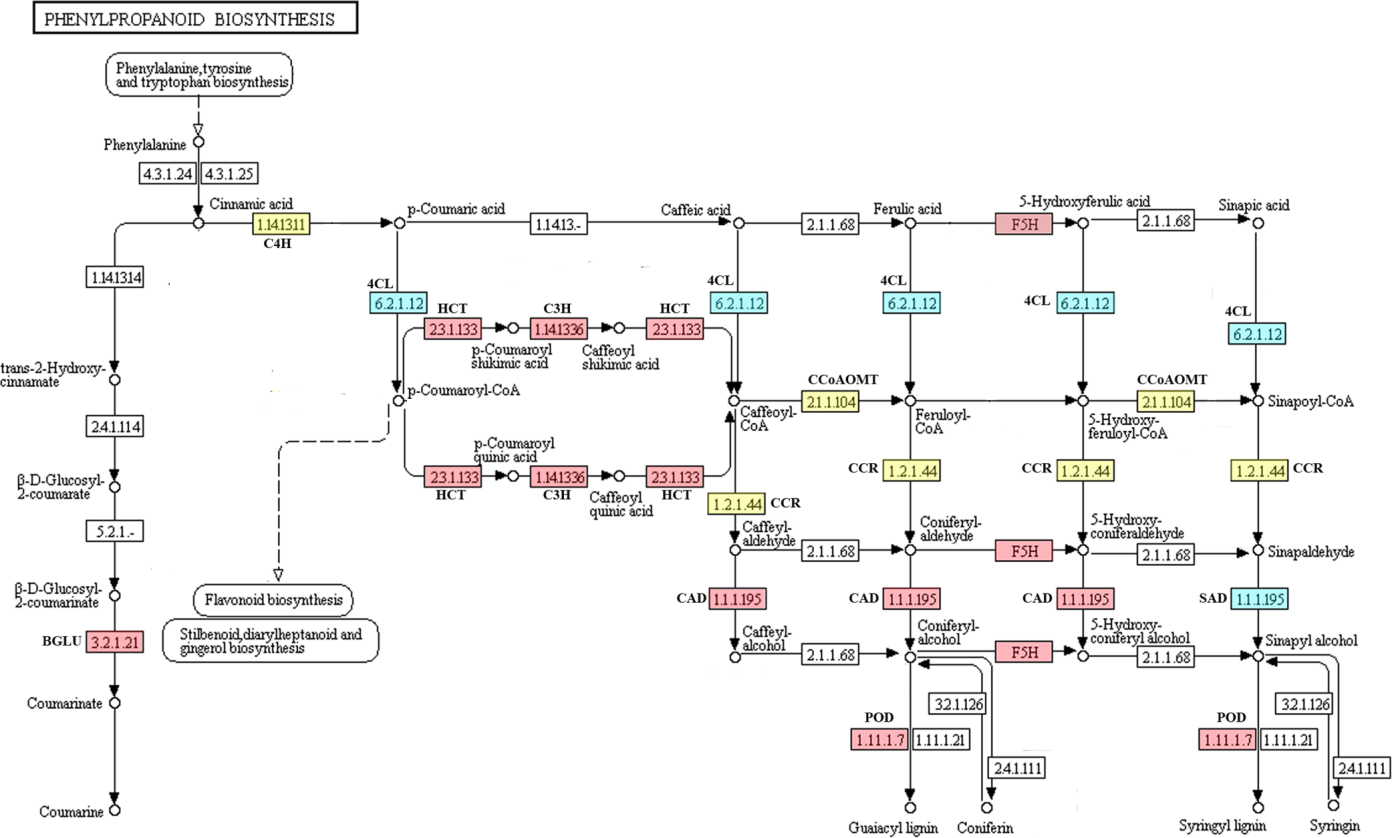
DEGs related to phenylpropanoid metabolism at 55 DAF in CD and CL fruits. 23.1.133, Shikimate O-hydroxycinnamoyltransferase (HCT); F5H, Ferulate 5-hydroxylase (F5H); 1.14.1336, Coumaroylquinate 3-monooxygenase (C3H); 1.2.1.44, Cinnamoyl-CoA reductase (CCR); 6.2.1.12, 4-Coumarate-CoA ligase (4CL); 21.1.104, Caffeoyl-CoA O-methyltransferase (CCoAOMT); 1.14.1311, Cinnamic acid 4-hydroxylase (C4H); 1.11.1.7, Peroxidase (POD); 1.1.1.195, Cinnamyl alcohol dehydrogenase/Sinapyl alcohol dehydrogenase (CAD/SAD); 3.2.1.21, β-Glucosidase (BGLU). According to the transcriptome data analysis, red indicates up-regulated genes, blue indicates down-regulated genes, and yellow indicates genes with no significant difference in expression (CD55 vs. CL55).

Based on the transcriptome profiles from CD23 vs. CL23, CD55 vs. CL55 and CD145 vs. CL145, some DEGs encoding key enzymes involved in phenylpropanoid metabolism were identified (Table 3, S1 and S2 Figs), such as *β-glucosidase* (*BGLU*) (1), *C4H* (1), *4CL* (1), *HCT* (1), *C3H* (1), *CCoAOMT* (1), *CCR* (1), *F5H* (2), *CAD* (2), *SAD* (1) and *POD* (3) (Table 3, Fig 6). Among them, β-glucosidase is the key enzyme involved in the synthesis of coumarin (K01188). Cinnamic acid is the product of PAL and a shared precursor of the coumarin and lignin biosynthesis pathways (Fig 6), and the remaining 14 genes are key synthetic genes involved in lignin biosynthesis [11,12].

**Table 3:** DEGs related to phenylpropanoid metabolism in CD and CL fruits.

Gene Name	Gene ID	Genome ID	23 DAF FPKM		55 DAF FPKM		145 DAF FPKM	
			CD	CL	CD	CL	CD	CL
***C4H***	pyrus_GLEAN_10019526	Pbr017290.1	148.630	248.149	1105.056	1112.448	93.949	110.414
***4CL***	pyrus_GLEAN_10022547	Pbr012851.1	0.550	1.115	7.640	9.777	0.500	0.257
***HCT***	pyrus_GLEAN_10018682	Pbr018314.1	0.178	1.528	4.246	3.293	0.039	0.083
***C3H***	pyrus_GLEAN_10037033	Pbr020891.1	0.000	0.000	13.280	7.224	0.000	0.000
***CCoAOMT***	pyrus_GLEAN_10008165	Pbr034039.1	7.340	19.810	887.390	856.848	5.320	5.882
***CCR***	pyrus_GLEAN_10036516	Pbr022402.1	47.870	54.873	412.260	362.289	38.460	28.952
***F5H***	pyrus_GLEAN_10004521	Pbr040547.1	0.486	1.012	78.400	57.626	0.493	0.254
	pyrus_GLEAN_10016369	Pbr022142.1	1.049	1.275	94.332	65.987	0.741	0.297
***CAD***	pyrus_GLEAN_10013164	Pbr026287.1	0.190	0.507	70.180	57.988	0.360	0.308
	CUFF10.308.2	Pbr006899.1	0.110	0.317	4.060	1.355	0.080	0.088
***SAD***	pyrus_GLEAN_10027829	Pbr004675.1	0.000	0.000	1.640	14.399	0.000	0.000
***POD***	pyrus_GLEAN_10007497	Pbr035186.1	24.932	94.141	443.889	294.841	74.683	71.264
	pyrus_GLEAN_10034103	Pbr031894.1	0.300	27.366	3798.167	2504.342	448.160	433.497
	pyrus_GLEAN_10007933	Pbr034480.1	0.000	0.000	1.912	0.572	0.000	0.000
***BGLU***	pyrus_GLEAN_10039412	Pbr020361.1	0.835	1.878	38.207	35.540	1.457	1.075

FPKM values were obtained by deep sequencing analysis.

**Fig 7:**
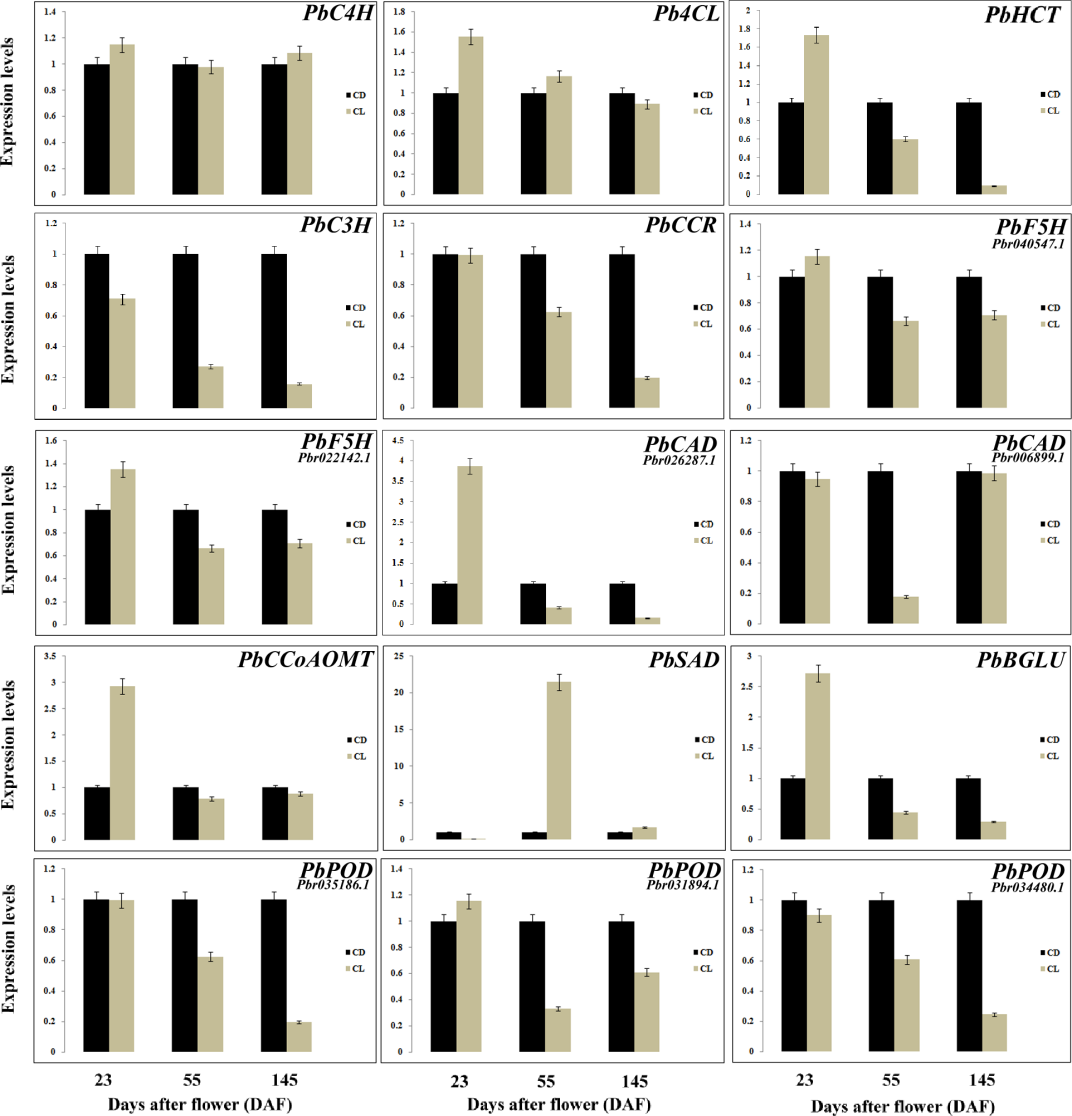
Expression analysis of 15 DEGs related to phenylpropanoid biosynthesis in pear as assessed by qRT-PCR.

As shown in Table 3, most genes involved in lignin biosynthesis were expressed at lower levels in CD fruits compared with CL at 23 DAF, which is consistent with qRT-PCR results (Fig 7). Only the expression levels of *C3H*, *SAD*, *CAD* (Pbr006899.1) and *POD* (Pbr034480.1) were significantly increased in CD23 compared with CL23. These results indicate that lignin biosynthesis is more active in CL fruits compared with CD fruits during the initial stage of fruit development.

According to our research, the lignin content in CD and CL fruits peaked at 55 DAF, whereas the stone cell content maintained an upward trend until 63 DAF. Therefore, DEGs from CD and CL fruits at 55 DAF were selected for further analysis. As noted in Fig 7 and S2 Table, *C4H* and *CCoAOMT* expression exhibited no apparent difference between CD55 and CL55 fruits, indicating that the low contents of stone cells and lignin in ‘Lianglizaosu’ may not be caused by these two genes. Additionally, the expression levels of *HCT*, *C3H*, *F5H*, *CCR*, *CAD* and *POD*, which are genes located in the middle and downstream of lignin biosynthesis, were increased in CD55 fruits compared with CL55 fruits, suggesting important roles in determining stone cell and lignin levels (Fig 7, Table 3). The results indicate that the differences in stone cell and lignin contents between ‘Lianglizaosu’ and ‘Dangshan Su’ were mainly due to differential expression of lignin synthase genes located in the middle and downstream of lignin biosynthesis (Fig 6).

The results of qRT-PCR showed that in mature pear fruit, the *4CL*, *CCR* and *F5H* expression levels in CD145 were increased compared with CL145, consistent with the transcriptome results (Table 3, Fig 7). The transcriptome results revealed no significant difference in *C4H*, *CCoAOMT*, *CAD* and *POD* expression between CD and CL fruits at 145 DAF (Table 3, S2 Table). qRT-PCR analysis revealed that only *C4H* expression in CL145 was slightly increased compared with CD145, whereas *CCoAOMT*, *CAD* and *POD* expression was increased in CD145 compared with CL145 (Fig 7). Overall, although lignin metabolism in CD and CL fruits was stable at the fruit ripening stage, lignin synthesis was still active in CD fruits compared with CL fruits.

Therefore, lignin synthesis in CL fruits was enhanced compared with CD fruits at the early stage of fruit development and was weaker than that in CD fruits in the middle and later stages of fruit development, resulting in the lower lignin and stone cell contents in mature fruit.

### Discovery of novel genes involved in stone cell development

**Fig 8:**
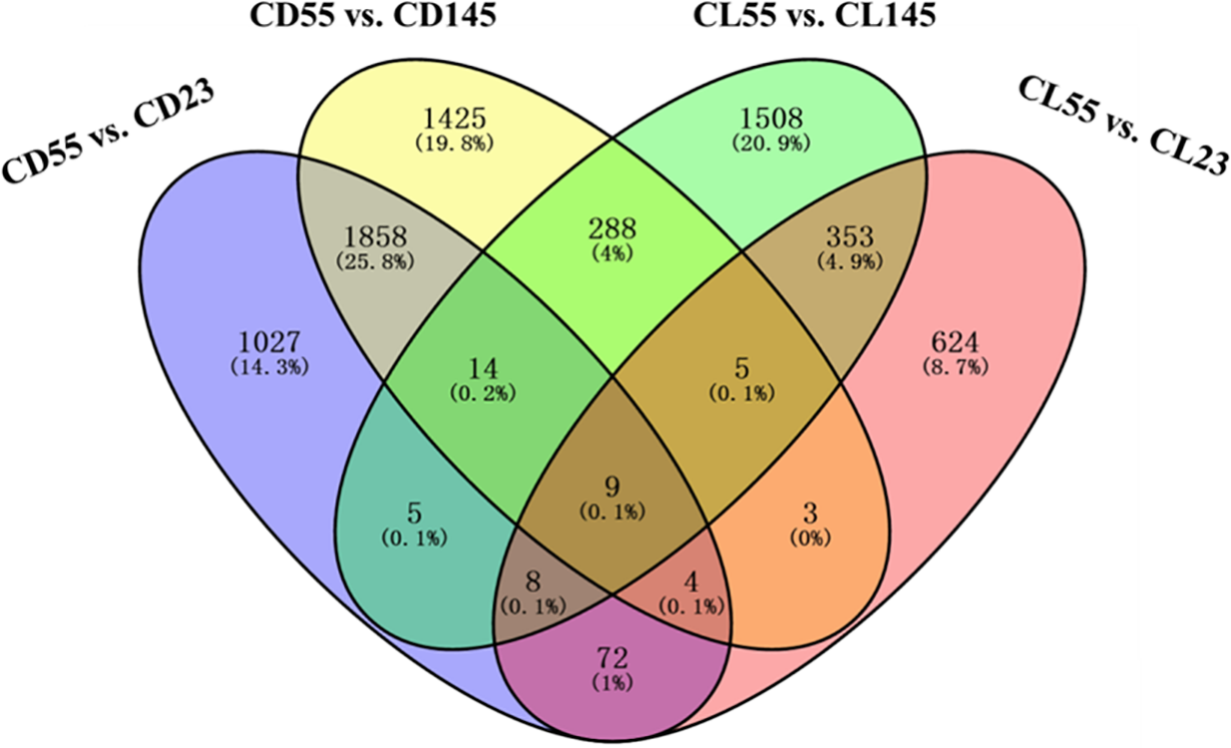
Venn diagram analysis of stone cell development-related DEGs.

To further explore the candidate genes that may be involved in stone cell development, crossover analysis of six transcriptome datasets was performed. The results revealed 1027 differentially expressed genes between 23 DAF and 55 DAF and 1425 differentially expressed genes between 55 DAF and 145 DAF over the course of ‘Dangshan Su’ pear fruit development (Fig 8). In addition, 624 differentially expressed genes were identified between 23 DAF and 55 DAF, and 1508 differentially expressed genes were identified between 55 DAF and 145 DAF in ‘Lianglizaosu’ pear fruits. However, Venn diagram analysis revealed that only 9 genes were common among the four comparisons (Fig 8). These genes included BON1-associated protein (BAP), nudix hydrolase (NUDT), pentatricopeptide repeat (PPR) protein, probable protein phosphatase 2C 25 (PP2C25), F-box protein (FBX) and 2 uncharacterized proteins. As shown in Table 4 and S3 Table, these 9 genes were up-regulated in ‘Dangshan Su’ pear, and their expression was considerably increased compared with ‘Lianglizaosu’ at 55 DAF, which is the peak period of stone cell formation, suggesting that these genes likely play an important role in stone cell development.

**Table 4:** DEGs related to stone cell development in different pear varieties at different developmental stages.

Gene name	Gene ID	23 DAF FPKM		55 DAF FPKM		145 DAF FPKM	
		CD	CL	CD	CL	CD	CL
**uncharacterized (LOC103946016)**	CUFF38.82.1	0.000	0.762	2.475	0.001	0.000	2.135
**probable protein phosphatase 2C 25 (LOC103931233)**	CUFF51.591.1	4.639	38.343	109.765	5.324	12.242	26.472
**BON1-associated protein 2-like (LOC103932710)**	pyrus_GLEAN_10009492	0.942	18.567	91.055	3.033	13.006	38.099
**BON1-associated protein 2-like (LOC103932711)**	pyrus_GLEAN_10009493	0.837	4.549	67.178	0.000	0.370	4.300
**uncharacterized (LOC103930249)**	pyrus_GLEAN_10011690	0.981	2.918	14.379	0.125	1.892	2.626
**F-box protein (LOC103959390)**	pyrus_GLEAN_10023367	0.038	3.754	2.621	0.000	0.021	1.568
**pentatricopeptide repeat-containing protein (LOC103958771)**	pyrus_GLEAN_10024043	0.006	2.175	2.956	0.016	0.531	2.583
**nudix hydrolase 17 (LOC103950554)**	pyrus_GLEAN_10031740	25.760	117.173	218.495	11.397	53.575	92.233
**nudix hydrolase 18 (LOC103946908)**	pyrus_GLEAN_10035206	5.413	44.473	49.584	1.418	7.251	9.688

FPKM values were obtained by deep sequencing analysis.

**Fig 9:**
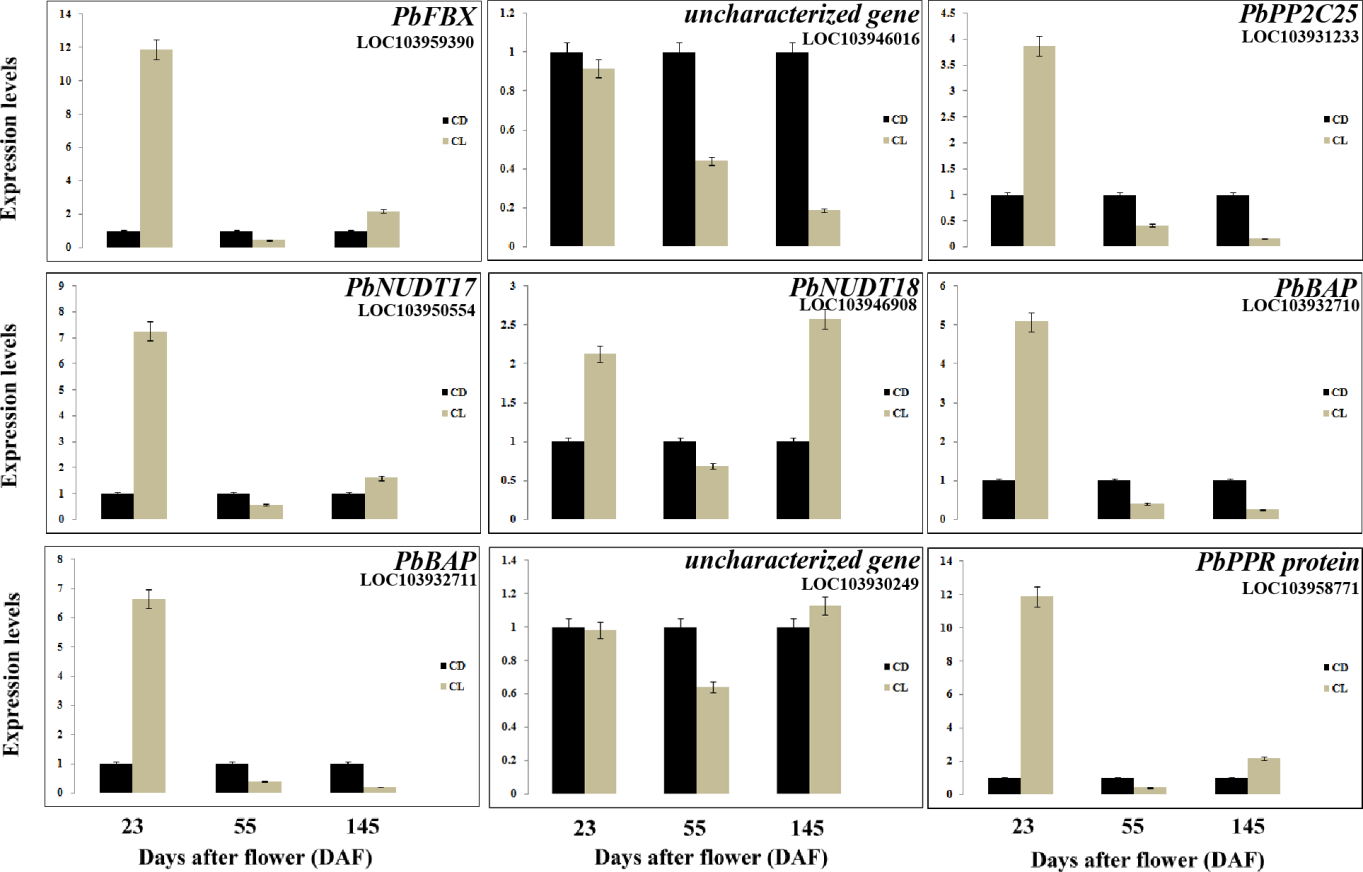
qRT-PCR validation of the expression level of putative novel genes related to stone cell development.

To validate the transcriptome data, the relative expression levels of 9 selected genes were analyzed by qRT-PCR (Fig 9), and most of these genes exhibited consistent results with the transcriptome data, indicating that the transcriptome analysis is reliable. The expression patterns of the 9 genes were divided into the following four categories. The first category included those with increased expression levels in CD fruits compared with CL fruits across pear development, such as uncharacterized gene LOC103946016. The second category included genes with increased expression in CD55 and CD145 compared with CL55 and CL145, such as the genes encoding PP2C25 (LOC103931233) and BAPs (LOC103932710, LOC103932711). The third category included genes with increased expression in CD compared with CL fruits only at 55 DAF, such as the genes encoding FBX (LOC103959390), PPR protein (LOC103958771) and NUDTs (LOC103950554, LOC103946908). The final category included an uncharacterized gene (LOC103930249) with increased expression in CD23 and CD55 compared with CL23 and CL55.

### Subcellular localization of candidate lignin metabolism genes

**Fig 10:**
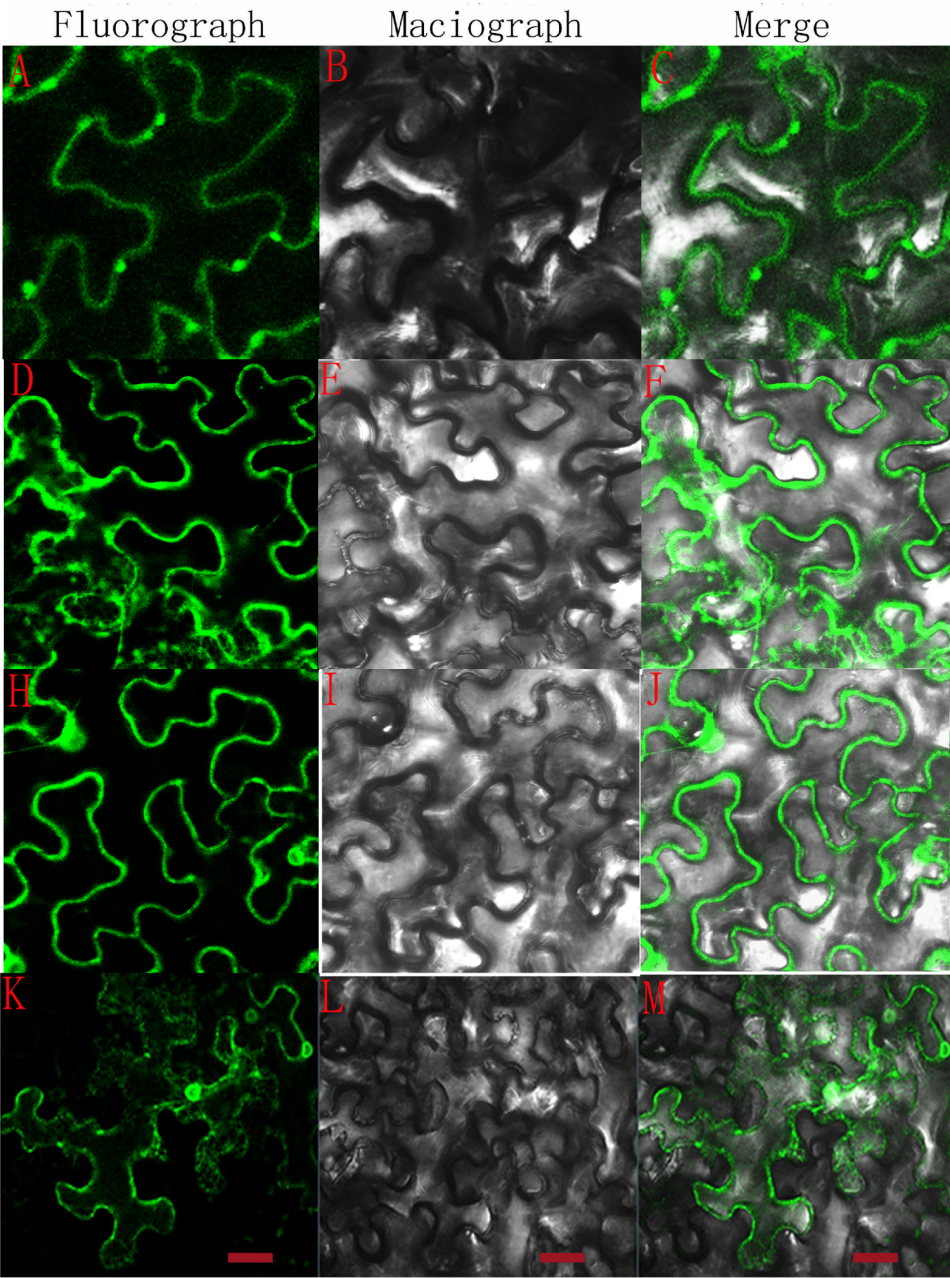
Subcellular localization of candidate lignin metabolism genes. **(**A-C): pCAMBIA1304-PbF5H-GFP; (E-G): pCAMBIA1304-PbC3H-GFP; (H-J): pCAMBIA1304-PbPOD-GFP; (K-M): pCAMBIA1304-GFP (bar = 20 μm).

The CDS of three differentially expressed genes (*PbC3H*, *PbF5H* and *PbPOD*) were inserted into the vector pCAMBIA1304-GFP. Then, the expression of these three fusion proteins was observed by transient transformation mediated by *Agrobacterium* EHA105. Green fluorescence was detected in the plasma membrane, cytoplasm and nucleus of *Nicotiana benthamiana* leaf epidermis cells that were transformed with the empty vector pCAMBIA1304-GFP. However, the subcellular localization patterns of PbC3H-GFP, PbF5H-GFP and PbPOD-GFP were obviously different compared with the empty plasmid. C3H and F5H are key enzymes of lignin monomer synthesis, whereas POD is responsible for the polymerization of lignin monomers. The results of this study suggest that PbF5H-GFP and PbPOD-GFP are mainly localized to the plasma membrane, whereas PbC3H-GFP was detected in both the cytoplasm and plasma membrane (Fig 10). The locations of these three genes are consistent with their functions because lignin monomers are synthesized in the cytoplasm, and oxidation polymerization occurs when monomers are transported to the cell membrane and deposited at the SCW [12,37,38].

## Discussion

Numerous recent studies have focused on lignin metabolism and made significant progress toward understanding this important process [12,39]. However, the effects of lignin metabolism-related genes on stone cell development are unclear. Here, comparative transcriptome analysis was performed to identify DEGs from the three different developmental stages of ‘Dangshan Su’ and ‘Lianglizaosu’. Comparative transcriptional and qRT-PCR analysis demonstrated that the stone cell formation in pear flesh was related to a branch of phenylpropanoid metabolism, namely, lignin metabolism. As shown in Fig 4, the fruit of ‘Lianglizaosu’ has a lower stone cell and lignin content than that of ‘Dangshan Su’. However, the dynamic changes in the contents of stone cells and lignin within both varieties exhibited similar trends. Correlation analysis uncovered a close relationship between stone cell and lignin contents (Table 1). Moreover, changes in the expression of lignin-related genes in different developmental stages were consistent with the trends of stone cell and lignin contents in the fruits of ‘Lianglizaosu’ and ‘Dangshan Su’ (S4 Table), indicating that lignin metabolism genes can affect the development of stone cells by regulating lignin synthesis.

Comparative transcriptome data and qRT-PCR results revealed that the transcript levels of lignin biosynthetic unigenes encoding C3H, HCT, F5H, CAD and POD were significantly increased in CD55, whereas the transcript level of the unigene encoding SAD was significantly increased in CL55 (Fig 7, Table 3, S2 Table). SAD is a key enzyme involved the synthesis of S-lignin precursors (sinapyl alcohol) [40–42]. Thus, increased SAD levels suggest increased S-lignin biosynthesis in ‘Lianglizaosu’ compared with ‘Dangshan Su’. Yan et al. (2014) reported that an increased ratio of G-lignin to S-lignin results in a more stable lignin polymer and more condensed groups of stone cells [13]. *SAD* expression up-regulation in ‘Lianglizaosu’ may lead to a reduced G/S ratio, making the stone cells less prone to highly polymerized lignin and reducing the size of stone cell clusters in ‘Lianglizaosu’ compared with ‘Dangshan Su’ pear.

In addition, most of the identified 14 key enzyme genes in lignin synthesis were significantly up-regulated in both ‘Dangshan Su’ and ‘Lianglizaosu’ at 55 DAF compared with fruits at 23 and 145 DAF. However, the degree of up-regulation differed (S4 Table). From 23 DAF to 55 DAF, the upward trend of these genes (*C4H*, *4CL*, *HCT*, *C3H*, *CCoAOMT*, *CCR*, *F5H*, *CAD* and *POD*) in the ‘Dangshan Su’ pear was significantly higher than those in ‘Lianglizaosu’. For example, the expression levels of *HCT* (pyrus_GLEAN_10018682), *CCoAOMT* (pyrus_GLEAN_10008165) and *POD* (pyrus_GLEAN_10034103) were increased by 11.5 fold, 2.7 fold and 138 fold, respectively, compared with ‘Lianglizaosu’ (CD55/CD23 ratio divided by CL55/CL23 ratio) (S4 Table). This finding indicates that lignin metabolism-related gene expression was significantly increased to promote lignin synthesis and stone cell development at 55 DAF in ‘Dangshan Su’. These findings are consistent with the research conclusions of Cao et al. (2016) [43].

Lignin is a major carbon sink in the biosphere, accounting for approximately 30% of the greater than 1.4 × 10^12^ kg of carbon sequestered in terrestrial plant material each year [44]. Here, many carbon metabolism-related genes were also differentially expressed in CD and CL fruits at the same development stages, including genes involved in the reductive citrate cycle, the Calvin cycle, glycolysis, galactose degradation and the glucuronate pathway (S5 Table).

Given that stone cells are completely developed at 67 DAF [4], we mainly focused on developmental gene expression at 23 DAF and 55 DAF. According to the results of transcriptome sequencing and qRT-PCR, we found that genes for the key enzymes in the reductive citrate cycle (fumarate hydratase, FH and isocitrate dehydrogenase, IDH) were up-regulated at 23 DAF, whereas Calvin cycle (glyceraldehyde-3-phosphate dehydrogenase, GAPDH and sedoheptulose-1,7-bisphosphatase, SBPASE) and glucuronate pathway (UDP-glucose 6-dehydrogenase, UGDH and sorbitol dehydrogenase-like, SDH-like) genes were up-regulated at 55 or 145 DAF. Notably, genes involved in galactose degradation (Bifunctional UDP-glucose 4-epimerase and UDP-xylose 4-epimerase, UGE) and glycolysis (ATP-dependent 6-phosphofructokinase, ATP-PFK) were more highly expressed in CD fruits than in CL fruits at both 23 and 55 DAF (S3 Fig and S5 Table). The reductive citrate cycle synthesizes sugars and other organic molecules, which subsequently enter glycolysis. Phosphoenolpyruvate (PEP) and erythrose-4-phosphate (E4P) are metabolic intermediates of glycolysis and the Calvin cycle [16,45,46], which are the precursors for the synthesis of L-Phe. L-Phe is the substrate of the key enzyme PAL in the first step of lignin synthase [46,47]. Therefore, these three pathways provide precursors for lignin synthesis [16,45,46,48], explaining the higher lignin and stone cell contents in ‘Dangshan Su’ compared with ‘Lianglizaosu’.

Galactose degradation (KEGG: M00632) and the glucuronate pathway (KEGG: M00014) produce uridine diphosphate glucose (UDPG) directly [46,49,50]. UDPG then serves as a sugar donor for UDP-glucuronosyltransferases (UGTs), which are the key enzyme involved in the glycosylation and transport of monolignols [51,52,53]. Therefore, these two pathways may affect the lignin monomer transport. In addition, we identified *BGLU* (pyrus_GLEAN_10039412) (Table 3) in the transcriptome database.The results of qRT-PCR were consistent with transcriptome analysis, the expression of this gene at 55 and 145 DAF in ‘Dangshan Su’ was increased compared with ‘Lianglizaosu’ (Fig 7, S2 Table). In addition, its expression was significantly up-regulated at 55 DAF in the two varieties fruits (Table 3). Numerous studies have demonstrated that BGLU catalyzes deglycosylation of monolignol glucosides, thereby releasing monolignols to participate in lignin polymerization [11,12,51,54]. Thus, we hypothesize that UGT and BGLU are responsible for glycosylation and deglycosylation, respectively, of monolignols in pear fruit, which affects lignin synthesis by regulating monolignol transport [51,53].

Based on our Venn diagram analysis, nine genes were identified that were specifically up-regulated in ‘Dangshan Su’ pear fruits compared with ‘Lianglizaosu’ at 55 DAF, indicating that these genes may play roles in generating more stone cells (Fig 8, Table 4). One of these genes, pyrus_GLEAN_10023367, is an *FBX* that may affect lignin metabolism via the regulation of PAL [55]. In addition, a PPR protein (pyrus_GLEAN_10024043) was involved in plant development and RNA metabolism [56]. Given that miRNA could affect lignin metabolism and stone cell development via the regulation of laccases, this result suggests that the PPR protein may interact with RNA to control stone cell development [2,57]. PP2C25 (CUFF51.591.1) and NUDTs (pyrus_GLEAN_10031740 and pyrus_GLEAN_10035206) play roles in phosphorus modification and the hydrolysis of phosphorus-containing substances, respectively [58,59]. Given that the key enzymes in lignin synthesis, O-methyltransferases (OMTs), are regulated via phosphorus modification, it is likely that these two genes regulate lignin synthesis by phosphorylation and dephosphorylation [60]. Interestingly, we also identified two genes categorized as *BAP*s (pyrus_GLEAN_10009492 and pyrus_GLEAN_10009493), which are involved in plant programmed cell death (PCD) [61]. Given that stone cell formation involves PCD [6], it is reasonable to hypothesize that BAPs might play roles in the formation of stone cells in pear fruits. The specific functions of these 9 genes will be further validated in the future. Taken together, our results provide useful information on the metabolic pathways involved in stone cell development.

## Conclusions

In summary, through comparative analysis of physiological tests and transcriptome and qRT-PCR analyses between two different pear varieties, the differences in stone cell development between ‘Dangshan Su’ and ‘Lianglizaosu’ (low stone cell content bud sport of ‘Dangshan Su’) can be summarized by the following three points. 1. Differences in lignin content and metabolism. Lignin was higher in CD fruits compared with CL fruits at all developmental stages. The expression of most structural genes located in the middle and downstream of lignin metabolism in the CD fruits was increased compared with CL fruits at 55 DAF and 145 DAF. Therefore, lignin metabolism in CD fruits was enhanced compared with CL fruits at the middle and later stages of fruit development. This feature is a key factor in the high stone cell content of the CD fruit. *PbSAD* expression in CD fruit was significantly reduced compared with CL fruit, suggesting that the G/S ratio of CD fruit was increased compared with CL fruit, resulting in lignin stability and difficulties in degrading and forming highly aggregated SCCs [2,13]. 2. Differences in carbon metabolism. Partial gene expression was up-regulated in glycolysis, the Calvin cycle, galactose degradation and the glucuronate pathway of CD fruits, and their metabolic intermediates, such as PEP, E4P and UDGP, can be used as precursors in lignin synthesis. Therefore, the carbon metabolism of CD fruits is enhanced compared with CL fruits, which can provide sufficient raw material for lignin metabolism and result in extensive stone cell formation by promoting the synthesis and deposition of lignin. This is also one of the reasons that the ‘Dangshan Su’ pear’s lignin and stone cell content are higher than that of ‘Lianglizaosu’. 3. Differences in the expression of various putative regulatory genes. Some regulatory genes (*FBX*, *BAP*, *NUDT*, *PPR* and *PP2C25*) that were hypothesized to be involved in phenylpropanoid biosynthesis, phosphorylation, miRNA and PCD were up-regulated in CD fruits, which may also contribute to stone cell development.

We also clarified which gene family members play a major role in lignin synthesis and stone cell formation in pear via transcriptome analysis. Our results not only provide a theoretical basis for the elucidation of the relationship between lignin metabolism and stone cell development but also laid a foundation for clarifying the molecular origin of low stone cell content bud sports.

## Supporting information

S1 Fig KEGG classification of putative functions of differentially expressed genes. (TIF)

S2 Fig COG classification of putative functions of differentially expressed genes. (TIF)

S3 Fig Expression analysis of 9 DEGs related to carbon metabolism in pear as assessed by qRT-PCR. (TIF)

S1 File The statistics of unigenes annotated by different databases. (XLSX)

S2 File Annotated species distribution. (XLSX)

S1 Table Primers used in this study. (DOC)

S2 Table Changes of phenylpropanoid metabolism related genes in CD and CL fuirts at the same developmental stages. (DOC)

S3 Table DEGs related to stone cells development in CD and CL fruits. (DOC)

S4 Table Changes of lignin metabolism related genes in CD and CL fuirts at the different developmental stages. (DOC)

S5 Table DEGs related to carbon metabolism in CD and CL fruits. (DOC)
