# Supplementary figures and images for: Comparison of the transcriptomic analysis between two Chinese white pear (Pyrus bretschneideri Rehd.) genotypes of different stone cells contents
Source: PLoS One. 2017 Oct 31;12(10):e0187114. doi: 10.1371/journal.pone.0187114 (PMC5663431; doi:10.1371/journal.pone.0187114)

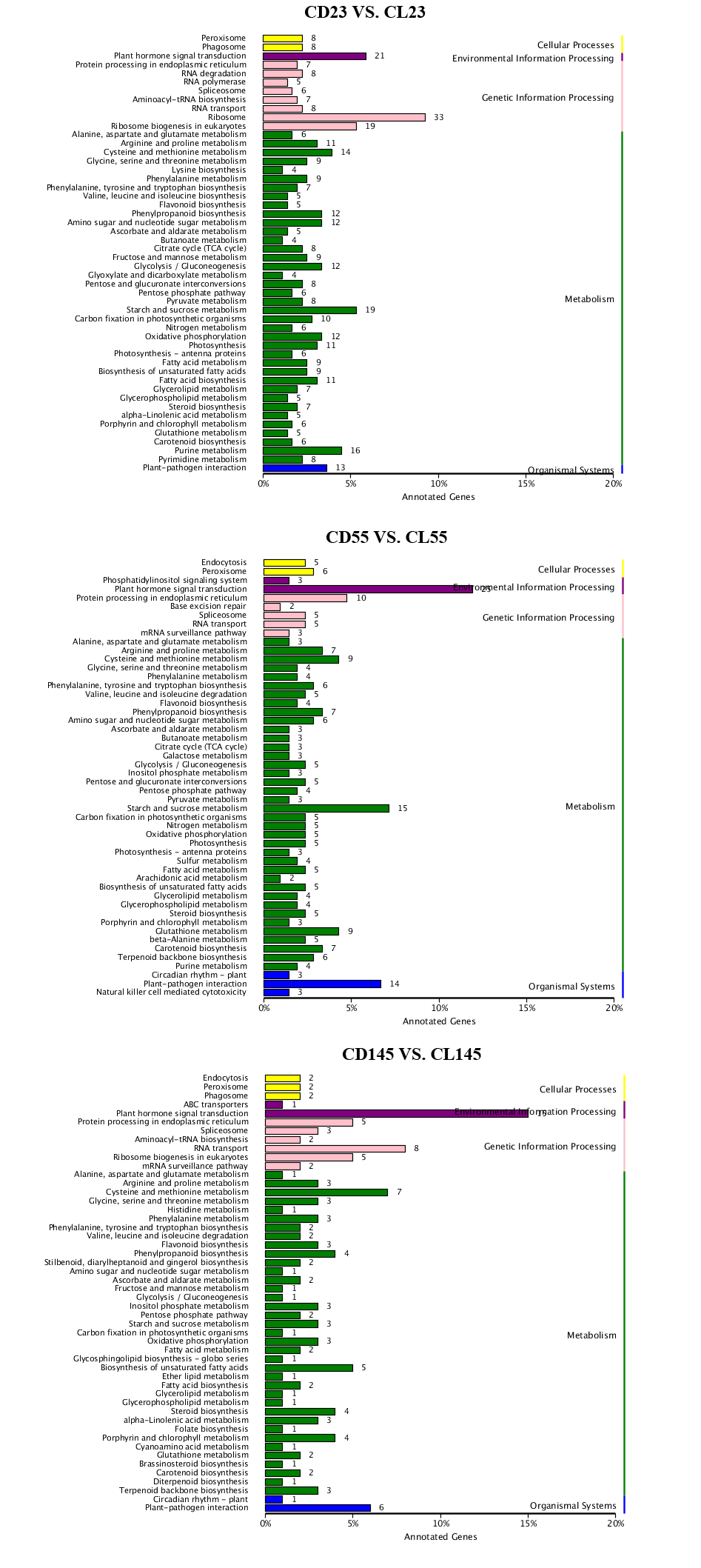

Supplement: S1 Fig — (TIF) [file pone.0187114.s001.tif]

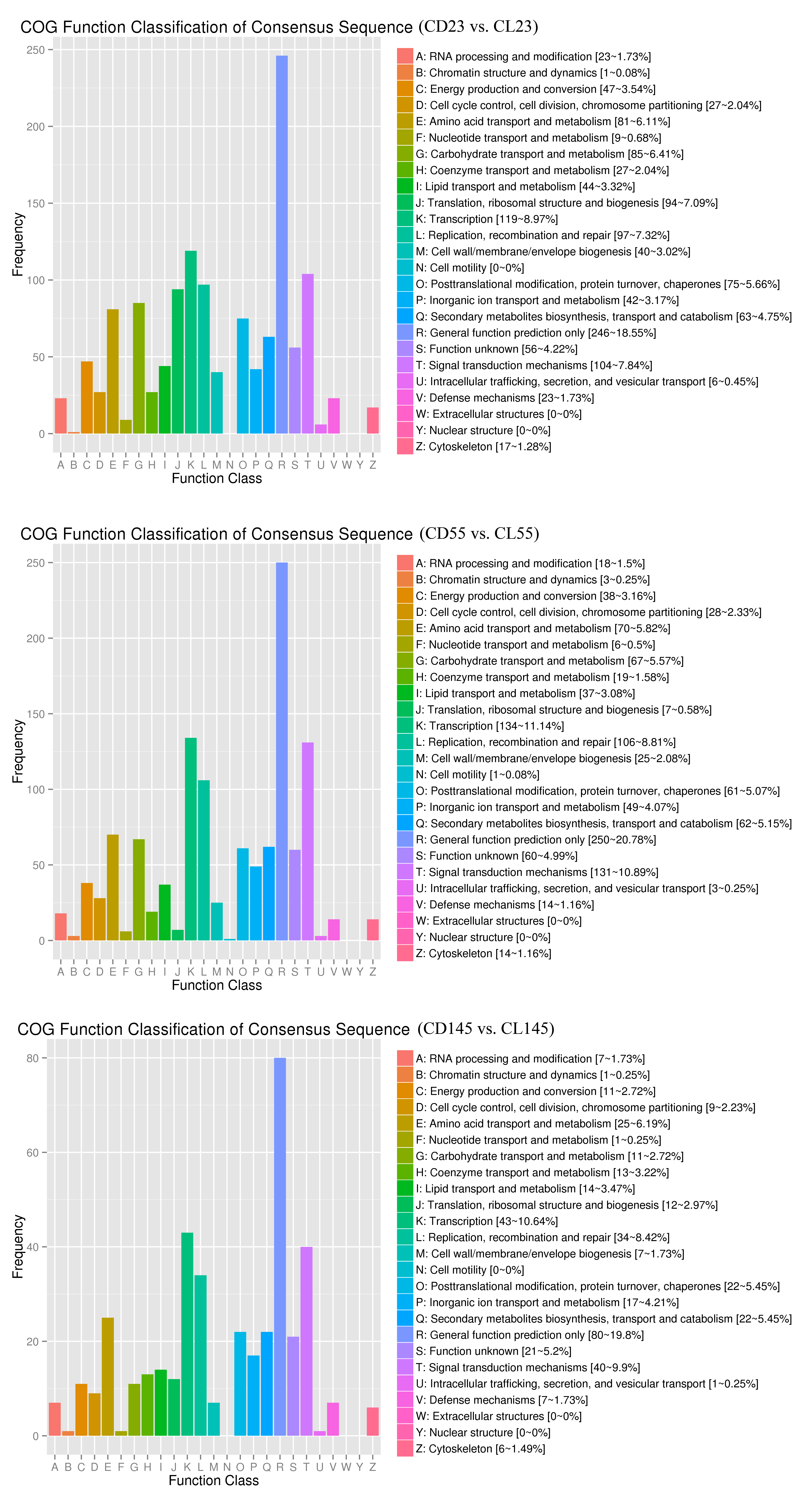

Supplement: S2 Fig — (TIF) [file pone.0187114.s002.tif]

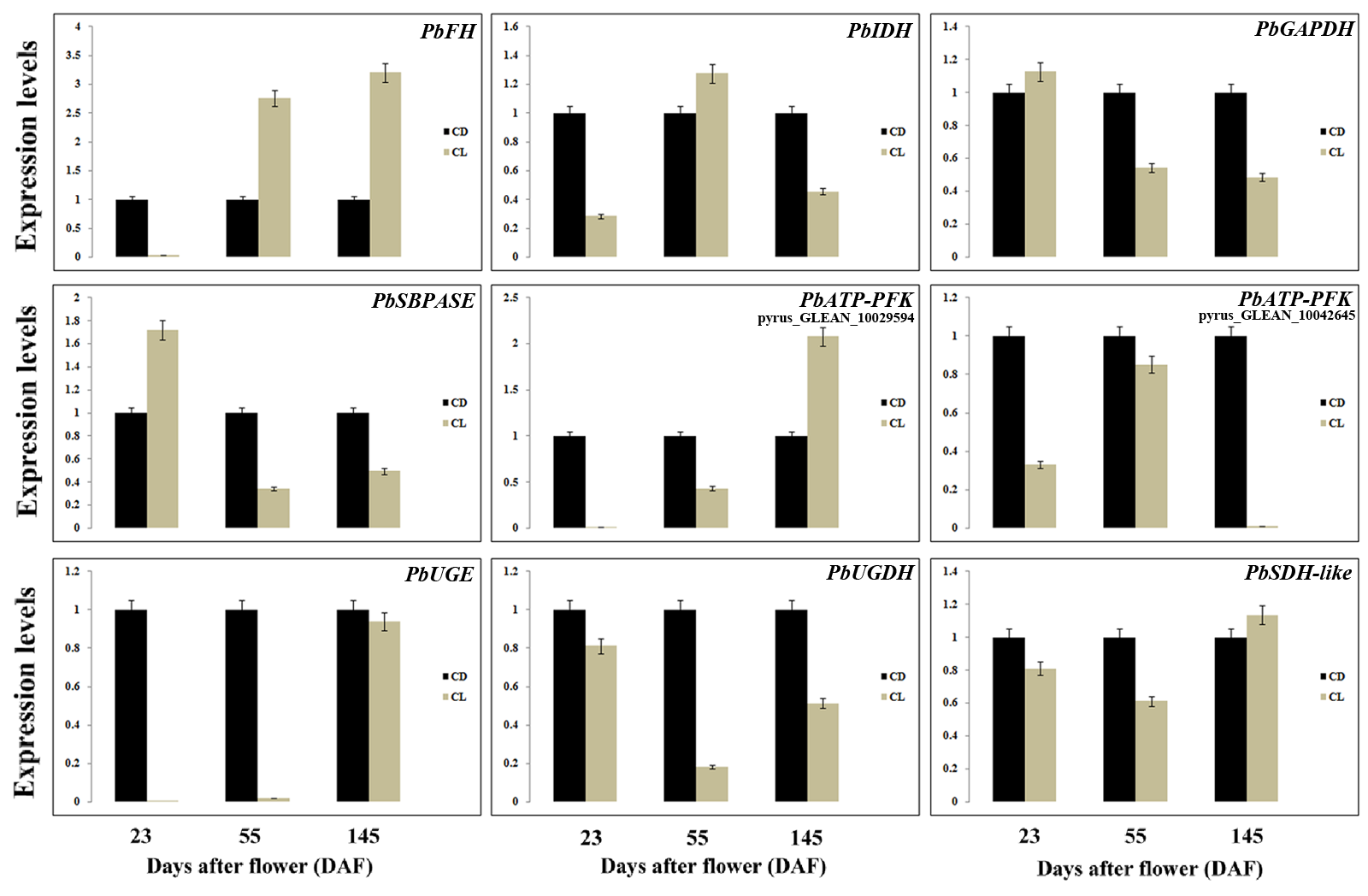

Supplement: S3 Fig — (TIF) [file pone.0187114.s003.tif]
