# Supplementary material for: Comparison of the transcriptomic analysis between two Chinese white pear (Pyrus bretschneideri Rehd.) genotypes of different stone cells contents
Source: PLoS One. 2017 Oct 31;12(10):e0187114. doi: 10.1371/journal.pone.0187114 (PMC5663431; doi:10.1371/journal.pone.0187114)
Supplement: S1 Table — (DOC) [file pone.0187114.s006.doc]

**Supporting information**

**S1 Table. Primers used in this study.**

| **Name** | **Transcriptome ID** | **Forward primers (5’-3’)** | **Reverse primers (5’-3’)** |
| --- | --- | --- | --- |
| **Primers used in qRT-PCR for validation of DEGs** | | | |
| **Uncharacterized gene** | CUFF38.82.1 | TCTGTGGAAGGAGATGGCTGG | AACTCGTTTCGTGCGACTACC |
| ***PbPP2C25*** | CUFF51.591.1 | GGGAAGACGAGAAAGCCAGAA | CTCCAAACACCACGGCATAAA |
| ***PbBAP*** | pyrus_GLEAN_10009492 | GACCGACACCAACAGCCAG | CCTTACCCCCTTGTGATTCCTC |
|  | pyrus_GLEAN_10009493 | ACACCAACAGCCAGTTTCGC | CCCCCTTGTAATCTCTCAGCC |
| **Uncharacterized gene** | pyrus_GLEAN_10011690 | TTCTGAACTGGAAACTCGGCG | CCAAAAGGGAACAGGGCAAT |
| ***PbFBX*** | pyrus_GLEAN_10023367 | CCGCTTCAAATGCGTGTCAA | TGGGGTGGGCAAGTAGTCAA |
| ***PbPPR*** | pyrus_GLEAN_10024043 | CCTTGGTCGTTCCAGGCAGT | CACGGTCAGGTTCACATCCC |
| ***PbNUDT17*** | pyrus_GLEAN_10031740 | CCCTCCGTATCTGAGTTATCTGG | GTTCTTGAAATCGGGGTCGTC |
| ***PbNUDT18*** | pyrus_GLEAN_10035206 | GGGAGGTTGGGAAATGGAT | GCCCTGGCTTTTGCTCTTGT |
| ***PbC4H*** | pyrus_GLEAN_10019526 | CGTTCATAAACTTCCCTACCTGC | AGTCGTTCCCGTTAGCCTCC |
| ***PbF5H*** | pyrus_GLEAN_10016369 | CAAGAACTTGCTGACGTGGT | TCTCGTGGAGGAGTAGTGGA |
|  | pyrus_GLEAN_10004521 | GTTTGGACTCACCGCTCCGAGAGCG | TGCACACTGTTTCTCTCTCCTCAAT |
| ***Pb4CL*** | pyrus_GLEAN_10022547 | TAAAATGAAGGATGAGGCGGCTGGA | TCCTTCAAAATGGCAGGGCATCACT |
| ***PbCCR*** | pyrus_GLEAN_10036516 | CAGTCCCTACAAAACAAGAACAAG | CGTGCCCTTTCCTACAAGC |
| ***PbCCoAOMT*** | pyrus_GLEAN_10008165 | TACATCAACTACCACAAGAG | CACAGACCCATTCCATAG |
| ***PbCAD*** | pyrus_GLEAN_10013164 | AAGGAAACTGAGGAGATGCTTGAAT | TACTTTATTAAATAAGATTGCTGCCG |
|  | CUFF10.308.2 | AGCTTGTAGGGGGAAGTGACATAGG | GCTTGCTACATCGATCACAAACCGA |
| ***PbSAD*** | pyrus_GLEAN_10027829 | AGATACCGATTTGTCATTGATGT | TTCATTATTCACCTTCTTGAGTCT |
| ***PbC3H*** | pyrus_GLEAN_10037033 | AATTGATTGAAGACGTCCATGAGCA | CCAGTGTTGCTCATGGACGTCTTCA |
| ***PbPOD*** | pyrus_GLEAN_10007497 | TCATCTTATTCTAGTGTGGTC | GGCGTAGTAGTTTGTTTTG |
|  | pyrus_GLEAN_10034103 | GACAGCAGTGCAATGTTGCCAATAA | CCTGACAAAGCTGCTTTTTGCTCTA |
|  | pyrus_GLEAN_10007933 | AAATGGGTCGGGCTAACG | TCTAGCTATTCACAACTGAACATT |
| ***PbHCT*** | pyrus_GLEAN_10018682 | AACCAATGATGGGAGTTTATCGCTG | TAACTCAAACCAGACTAAACTTCTC |
| ***PbBGLU*** | pyrus_GLEAN_10039412 | AAGAGATACCCAAAGATGT | AACTTGGACTAGAAGCAA |
| ***PbFH*** | pyrus_GLEAN_10015903 | TGCCAGAGAAGATGATTG | TCACCGTAGGATTTATGC |
| ***PbIDH*** | pyrus_GLEAN_10014589 | AATTCATTGACGCTGTTG | TCTTGTAGATTCGCATCA |
| ***PbGAPDH*** | pyrus_GLEAN_10016535 | AAAGGGTTGTTGATTTGG | AAGGAGAATAGGAGTAGGA |
| ***PbSBPASE*** | pyrus_GLEAN_10039550 | CTCCGACTAATCTTCCAA | AATCCAGTTCATATTCTATCTAC |
| ***PbATP-PFK*** | pyrus_GLEAN_10029594 | CTGAAGAGGATGTTGATGTG | AATGTTGAGTTGTGCTGTC |
|  | pyrus_GLEAN_10042645 | TACAGCAACGACGACAAG | ATCAATACAGAGAAGACTCAGAAG |
| ***PbUGE*** | pyrus_GLEAN_10005215 | AATTGGACGGAAGAAGAT | CAAGGAAGAACACAATGATT |
| ***PbUGDH*** | pyrus_GLEAN_10010645 | GTTGGATTCTTGGCTCAA | TTCAAGGAATCACTTATGCT |
| ***PbSDH-like*** | pyrus_GLEAN_10008826 | AGAAGGAGGTGGAAGAAG | TCCGAACCATTACAACTTG |
| ***Tubulin*** | internal reference | AGAACAAGAACTCGTCCTAC | GAACTGCTCGCTCACTCTCC |
| **Primers used in** **subcellular localization** | | | |
| ***PbF5H*** | pyrus_GLEAN_10004521 | GAAGATCTGATGGATTCTCTTCTGCAATCCTTGC | GACTAGTGAGTGGACAAACCACCCTTTTACTC |
| ***PbC3H*** | pyrus_GLEAN_10037033 | GAAGATCTGATGGCTGAGTTCATCAAGTACCCAA | GACTAGTATCAATTTCCATTGCGTCTATACAT |
| ***PbPOD*** | pyrus_GLEAN_10007497 | GAAGATCTGATGGGTCGATTTCATCTTATTCTAG | GACTAGTATTAAAAACACTGCAATCTCGACGA |
