# Supplementary material for: Comparison of the transcriptomic analysis between two Chinese white pear (Pyrus bretschneideri Rehd.) genotypes of different stone cells contents
Source: PLoS One. 2017 Oct 31;12(10):e0187114. doi: 10.1371/journal.pone.0187114 (PMC5663431; doi:10.1371/journal.pone.0187114)
Supplement: S2 Table — (DOC) [file pone.0187114.s007.doc]

**Supporting information**

**S2 Table. Changes of phenylpropanoid metabolism related genes in CD and CL fuirts at the same developmental stages.**

| **Gene Name** | **Gene ID** | **Genome ID** | **CD23/**  **CL23 ratio** | **CD55/**  **CL55 ratio** | **CD145/**  **CL145 ratio** |
| --- | --- | --- | --- | --- | --- |
| ***C4H*** | pyrus_GLEAN_10019526 | Pbr017290.1 | 0.599 | 0.993 | 0.851 |
| ***4CL*** | pyrus_GLEAN_10022547 | Pbr012851.1 | 0.489 | 0.781 | 1.946 |
| ***HCT*** | pyrus_GLEAN_10018682 | Pbr018314.1 | 0.116 | 1.289 | 0.470 |
| ***C3H*** | pyrus_GLEAN_10037033 | Pbr020891.1 | NA | 1.838 | NA |
| ***CCoAOMT*** | pyrus_GLEAN_10008165 | Pbr034039.1 | 0.370 | 1.036 | 0.904 |
| ***CCR*** | pyrus_GLEAN_10036516 | Pbr022402.1 | 0.872 | 1.138 | 1.328 |
| ***F5H*** | pyrus_GLEAN_10004521 | Pbr040547.1 | 0.480 | 1.360 | 1.941 |
|  | pyrus_GLEAN_10016369 | Pbr022142.1 | 0.823 | 1.430 | 2.495 |
| ***CAD*** | pyrus_GLEAN_10013164 | Pbr026287.1 | 0.374 | 1.210 | 1.169 |
|  | CUFF10.308.2 | Pbr006899.1 | 0.333 | 2.997 | 0.910 |
| ***SAD*** | pyrus_GLEAN_10027829 | Pbr004675.1 | NA | 0.114 | NA |
| ***POD*** | pyrus_GLEAN_10007497 | Pbr035186.1 | 0.265 | 1.506 | 1.048 |
|  | pyrus_GLEAN_10034103 | Pbr031894.1 | 0.011 | 1.517 | 1.034 |
|  | pyrus_GLEAN_10007933 | Pbr034480.1 | NA | 3.341 | NA |
| ***BGLU*** | pyrus_GLEAN_10039412 | Pbr020361.1 | 0.444 | 1.075 | 1.355 |

FPKM values were obtained by deep sequencing analysis. The ratio represents the fold change in the FPKM value in different development stages: a ratio ≥1.2 indicates genes that are up-regulated, a ratio ≤0.8 indicates genes that are down-regulated. Abbreviation: NA, not applicable.
