# Supplementary material for: Comparison of the transcriptomic analysis between two Chinese white pear (Pyrus bretschneideri Rehd.) genotypes of different stone cells contents
Source: PLoS One. 2017 Oct 31;12(10):e0187114. doi: 10.1371/journal.pone.0187114 (PMC5663431; doi:10.1371/journal.pone.0187114)
Supplement: S3 Table — (DOC) [file pone.0187114.s008.doc]

**Supporting information**

**S3 Table. DEGs related to stone cells development in CD and CL fruits.**

| **Gene name** | **Gene ID** | **Genome ID** | **Functional domain** | **CD23 /CL23 ratio** | | **CD55**  **/CL55**  **ratio** | | **CD145 /CL145 ratio** |
| --- | --- | --- | --- | --- | --- | --- | --- | --- |
| **uncharacterized (LOC103946016)** | CUFF38.82.1 | Pbr025308.1 | ZnF_PMZ | | 0.000 | | 3672.659 | 0.000 |
| **probable protein phosphatase 2C 25 (LOC103931233)** | CUFF51.591.1 | Pbr042867.1 | PP2Cc | | 0.121 | | 20.616 | 0.462 |
| **BON1-associated protein 2-like (LOC103932710)** | pyrus_GLEAN_10009492 | Pbr031668.1 | C2_SRC2_like | | 0.051 | | 30.019 | 0.341 |
| **BON1-associated protein 2-like (LOC103932711)** | pyrus_GLEAN_10009493 | Pbr031669.1 | C2_SRC2_like | | 0.184 | | NA | 0.086 |
| **uncharacterized (LOC103930249)** | pyrus_GLEAN_10011690 | Pbr028493.1 | zf-DHHC super family | | 0.336 | | 115.365 | 0.720 |
| **F-box protein (LOC103959390)** | pyrus_GLEAN_10023367 | Pbr011922.1 | F_box_assoc_1 | | 0.010 | | 15288.998 | 0.013 |
| **pentatricopeptide repeat-containing protein (LOC103958771)** | pyrus_GLEAN_10024043 | Pbr011501.1 | PPR | | 0.003 | | 185.325 | 0.206 |
| **nudix hydrolase 17 (LOC103950554)** | pyrus_GLEAN_10031740 | Pbr038831.1 | Nudix_Hydrolase super family | | 0.220 | | 19.171 | 0.581 |
| **nudix hydrolase 18 (LOC103946908)** | pyrus_GLEAN_10035206 | Pbr028358.1 | Nudix_Hydrolase super family | | 0.122 | | 34.959 | 0.748 |

The ratio represents the fold change in the FPKM value in different development stages: a ratio ≥1.2 indicates genes that are up-regulated, a ratio ≤0.8 indicates genes that are down-regulated. Abbreviation: NA, not applicable.
