# Supplementary material for: Comparison of the transcriptomic analysis between two Chinese white pear (Pyrus bretschneideri Rehd.) genotypes of different stone cells contents
Source: PLoS One. 2017 Oct 31;12(10):e0187114. doi: 10.1371/journal.pone.0187114 (PMC5663431; doi:10.1371/journal.pone.0187114)
Supplement: S4 Table — (DOC) [file pone.0187114.s009.doc]

**Supporting information**

| **Gene Name** | **Gene ID** | **CD55 /CD23 ratio** | **CD55/CD145 ratio** | **CL55 /CL23 ratio** | **CL55 /CL145 ratio** |
| --- | --- | --- | --- | --- | --- |
| ***C4H*** | pyrus_GLEAN_10019526 | 7.434946 | 11.7623 | 4.482984 | 10.07524 |
| ***4CL*** | pyrus_GLEAN_10022547 | 13.89091 | 15.28 | 8.76861 | 38.0428 |
| ***HCT*** | pyrus_GLEAN_10018682 | 23.85393 | 108.8718 | 2.155105 | 39.6747 |
| ***C3H*** | pyrus_GLEAN_10037033 | NA | NA | NA | NA |
| ***CCoAOMT*** | pyrus_GLEAN_10008165 | 120.8978 | 166.8026 | 43.25331 | 145.6729 |
| ***CCR*** | pyrus_GLEAN_10036516 | 8.612074 | 10.71919 | 6.602318 | 12.51344 |
| ***F5H*** | pyrus_GLEAN_10004521 | 161.3169 | 159.0264 | 56.94269 | 226.874 |
|  | pyrus_GLEAN_10016369 | 89.92564 | 127.3036 | 51.75451 | 222.1785 |
| ***CAD*** | pyrus_GLEAN_10013164 | 369.3684 | 194.9444 | 114.3748 | 188.2727 |
|  | CUFF10.308.2 | 36.90909 | 50.75 | 4.274448 | 15.39773 |
| ***SAD*** | pyrus_GLEAN_10027829 | NA | NA | NA | NA |
| ***POD*** | pyrus_GLEAN_10007497 | 17.80399 | 5.943642 | 3.131909 | 4.137306 |
|  | pyrus_GLEAN_10034103 | 12660.56 | 8.475025 | 91.5129 | 5.777069 |
|  | pyrus_GLEAN_10007933 | NA | NA | NA | NA |

**S4 Table. Changes of lignin metabolism related genes in CD and CL fuirts at the different developmental stages.**

FPKM values were obtained by deep sequencing analysis. The ratio represents the fold change in the FPKM value in different development stages: a ratio ≥1.2 indicates genes that are up-regulated, a ratio ≤0.8 indicates genes that are down-regulated. Abbreviation: NA, not applicable.
