# Supplementary material for: Comparison of the transcriptomic analysis between two Chinese white pear (Pyrus bretschneideri Rehd.) genotypes of different stone cells contents
Source: PLoS One. 2017 Oct 31;12(10):e0187114. doi: 10.1371/journal.pone.0187114 (PMC5663431; doi:10.1371/journal.pone.0187114)
Supplement: S5 Table — (DOC) [file pone.0187114.s010.doc]

**Supporting information**

**S5 Table. DEGs related to carbon metabolism in CD and CL fruits.**

| **Gene name** | | **Gene ID** |  | **CD23**  **FPKM** | **CD55 FPKM** | **CD145FPKM** | **CL23 FPKM** | **CL55 FPKM** | **CL145**  **FPKM** | **CD23 /**  **CL23 ratio** | **CD55/**  **CL55 ratio** |  |
| --- | --- | --- | --- | --- | --- | --- | --- | --- | --- | --- | --- | --- |
|  | **Reductive citrate cycle** | | | | | | | | | | | |
| **Fumarate hydratase** | | pyrus_GLEAN_10015903 |  | 43.07 | 0.40 | 8.68 | 13.957 | 0.783 | 11.251 | 3.086 | 0.508 |  |
| **Isocitrate dehydrogenase** | | pyrus_GLEAN_10014589 |  | 43.20 | 6.92 | 42.63 | 29.096 | 9.974 | 44.373 | 1.485 | 0.693 |  |
|  | **Reductive pentose phosphate cycle (Calvin cycle)** | | | | | | | | | | | |
| **Glyceraldehyde-3-phosphate dehydrogenase A** | | pyrus_GLEAN_10016535 |  | 85.82 | 14.27 | 1.13 | 148.403 | 10.441 | 0.472 | 0.578 | 1.367 |  |
| **Sedoheptulose-1,7-bisphosphatase** | | pyrus_GLEAN_10039550 |  | 19.50 | 3.10 | 0.54 | 21.105 | 2.139 | 0.152 | 0.924 | 1.448 |  |
|  | **Glycolysis** | | | | | | | | | | | |
| **ATP-dependent 6-phosphofructokinase** | | pyrus_GLEAN_10029594 |  | 15.46 | 2.92 | 11.49 | 9.098 | 2.819 | 18.199 | 1.699 | 1.037 |  |
|  | | pyrus_GLEAN_10042645 |  | 40.26 | 4.87 | 57.41 | 26.815 | 2.580 | 63.120 | 1.501 | 1.888 |  |
|  | **Galactose degradation (Leloir pathway)** | | | | | | | | | | | |
| **Bifunctional UDP-glucose 4-epimerase and UDP-xylose 4-epimerase** | | pyrus_GLEAN_10005215 |  | 1348.50 | 118.40 | 972.39 | 1101.915 | 57.147 | 921.012 | 1.224 | 2.072 |  |
|  | **Glucuronate pathway** | | | | | | | | | | | |
| **UDP-glucose 6-dehydrogenase 5** | | pyrus_GLEAN_10010645 |  | 0.00 | 2.79 | 0.15 | 0.000 | 0.001 | 0.000 | 0.000 | 2860.400 |  |
| **Sorbitol dehydrogenase-like** | | pyrus_GLEAN_10008826 |  | 0.05 | 8.82 | 0.08 | 0.279 | 0.941 | 0.506 | 0.181 | 9.370 |  |
|  | | pyrus_GLEAN_10008827 |  | 42.61 | 368.45 | 1784.84 | 51.758 | 215.436 | 1928.785 | 0.823 | 1.710 |  |
|  | | pyrus_GLEAN_10008831 |  | 16.81 | 86.26 | 381.72 | 116.364 | 70.359 | 420.947 | 0.144 | 1.226 |  |
|  | | pyrus_GLEAN_10008832 |  | 0.11 | 2.55 | 0.00 | 0.485 | 1.333 | 0.047 | 0.218 | 1.909 |  |

FPKM values were obtained by deep sequencing analysis. The ratio represents the fold change in the FPKM value in different development stages: a ratio ≥1.2 indicates genes that are up-regulated, a ratio ≤0.8 indicates genes that are down-regulated. Abbreviation: NA, not applicable.
